# Structure determination and analysis of titin A-band fibronectin type III domains provides insights for disease-linked variants and protein oligomerisation

**DOI:** 10.1016/j.jsb.2023.108009

**Published:** 2023-09

**Authors:** Martin Rees, Roksana Nikoopour, Alexander Alexandrovich, Mark Pfuhl, Luis R. Lopes, Mohammed M. Akhtar, Petros Syrris, Perry Elliott, Gerry Carr-White, Mathias Gautel

**Affiliations:** a Randall Centre for Cell and Molecular Biophysics, King’s College London BHF Centre of Research Excellence, United Kingdom; b School of Cardiovascular Sciences and Medicine, King’s College London, United Kingdom; c Guy's and St Thomas' NHS Foundation Trust, London, United Kingdom; d Institute of Cardiovascular Science, University College London, United Kingdom; e Barts Heart Centre, St Bartholomew’s Hospital, London, United Kingdom; f School of Biomedical Engineering and Imaging Sciences, Rayne Institute, King's College London, St Thomas' Hospital, London, United Kingdom

**Keywords:** Titin, Fibronectin type III, Muscle, Myopathy, X-ray crystallography

## Abstract

Titin is the largest protein found in nature and spans half a sarcomere in vertebrate striated muscle. The protein has multiple functions, including in the organisation of the thick filament and acting as a molecular spring during the muscle contraction cycle. Missense variants in titin have been linked to both cardiac and skeletal myopathies. Titin is primarily composed of tandem repeats of immunoglobulin and fibronectin type III (Fn3) domains in a variety of repeat patterns; however, the vast majority of these domains have not had their high-resolution structure determined experimentally. Here, we present the crystal structures of seven wild type titin Fn3 domains and two harbouring rare missense variants reported in hypertrophic cardiomyopathy (HCM) patients. All domains present the typical Fn3 fold, with the domains harbouring variants reported in HCM patients retaining the wild-type conformation. The effect on domain folding and stability were assessed for five rare missense variants found in HCM patients: four caused thermal destabilization of between 7 and 13 °C and one prevented the folding of its domain. The structures also allowed us to locate the positions of residues whose mutations have been linked to congenital myopathies and rationalise how they convey their deleterious effects. We find no evidence of physiological homodimer formation, excluding one hypothesised mechanism as to how titin variants could exert pathological effects.

## Highlights

• We here present the crystal structures of 7 wild type fibronectin type III domains from the massive sarcomeric protein titin.
• The crystal structures of two of these domains containing missense variants found in patients with the heart disease hypertrophic cardiomyopathy (HCM) were also determined and showed little difference to the wild type domains.
• Five missense variants found in patients with HCM were shown to destabilise their domains by 7.4–13.0 °C.
• Analysis of crystal contacts revealed no evidence for physiological domain dimerization.

## Graphical abstract

**Figure:**
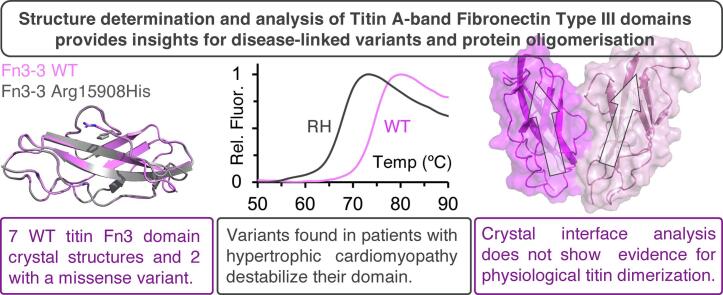

## 1 Introduction

Titin is a protein expressed in the skeletal and cardiac, or striated, muscle of vertebrates that plays various roles in muscle structure and function. Encoded by the gene *TTN*, titin spans half a sarcomere (the basic contractile unit of muscle) and has various functions including preventing sarcomere over-extension (Gautel and Djinović-Carugo, 2016), acting as a molecular ruler for thick filament formation (Tonino et al., 2017) and as a hub for numerous signalling molecules (Kruger and Linke, 2011, Linke and Hamdani, 2014).

**Fig. 1:**
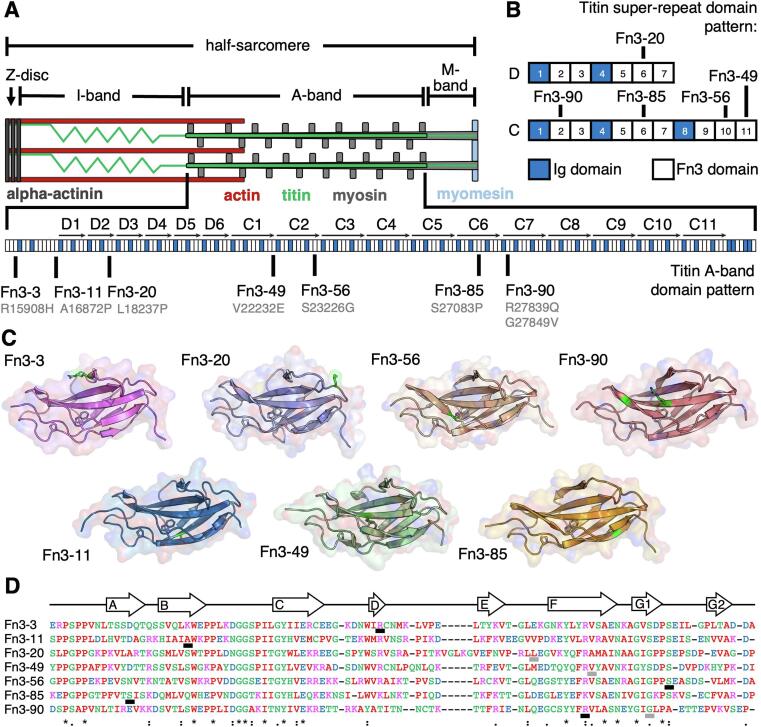
**Crystal structures of titin Fn3-3, Fn3-11, Fn3-20, Fn3-49, Fn3-56, Fn3-85 and Fn3-90. A:** Diagram showing main components of striated muscle half-sarcomere and domain structure of A-band region of Titin. Domain position of the crystal structures solved and the variants studied in this work are indicated. **B:** Diagram of Titin A-band D-zone and C-zone super-repeat domain pattern. Position of the studied domains in these super-repeats are indicated. **C:** Crystal structures of wild type Titin domains solved in this work shown in cartoon and surface representation. All structures are presented in the same orientation with their N- and C- termini on the left and right, respectively. Sidechains of residues conserved in all titin Fn3 domains are shown in a stick representation. Sidechains of residues with variants studied in this work are coloured green and shown in a stick representation. **D:** Structural sequence alignment of domains in this work with the following residue colour scheme: positively charged, magenta; negatively charged, blue; small and/or hydrophobic, red; polar and glycine, green. Conserved (*), strongly conserved (:) and weakly conserved (.) residues as defined in Clustal Omega are indicated. The consensus secondary structure of the domains is shown above the alignment. The residues mutated in patients in an HCM cohort, and the residues with variants previously linked to congenital myopathy are underlined in black and grey, respectively.

The sarcomere is a three-filament system in striated muscle where the bi-polar myosin-containing thick filaments, crosslinked by myomesin in the centre of the filament at the M−band, inter-digitate with actin-containing thin filaments which are cross-linked by alpha-actinin at the Z-disk (Fig. 1A). Movement is created by the ATP-powered movement of myosin heads along the thin filaments, which results in shortening of the sarcomere and therefore muscle contraction (Huxley, 1969). The third filament is titin, which provides permanent, physical links between the thick and thin filaments.

Titin is anchored to the Z-disk at its N-terminus via interactions with telethonin (Zou et al., 2006) and alpha-actinin (Ohtsuka et al., 1997, Young et al., 1998), and traverses the I-band where it acts as a molecular spring (Linke et al., 2002). Along the A-band, titin is associated with the thick filament, and reaches the M−band at its C-terminus where the protein is tethered via interactions with obscurin, obscurin-like 1 (Fukuzawa et al., 2008) and possibly myomesin (Obermann et al., 1997). Titin also binds to regulatory proteins including those involved in autophagy (Bogomolovas et al., 2021, Lange et al., 2005), ubiquitination (Centner et al., 2001, Mrosek et al., 2007) and phosphorylation (Tharp et al., 2020, Yamasaki et al., 2002) to help maintain myocyte homeostasis.

Titin is the largest protein discovered, with a molecular weight of ∼ 3MDa, and has a largely repetitive, modular structure. Depending on the transcript expressed, the protein is composed of tandem repeats of up to 169 immunoglobulin (Ig) and 132 fibronectin type-III (Fn3) domains, unique regions and a protein kinase. Based on the inferred complete (IC) sequence of titin, which includes all possible residues expressed in titin but does not correspond to an expressed transcript, Fn3 domains comprise ∼ 36% of the total sequence. All Fn3 domains are expressed in all full-length transcripts and comprise a greater part of the primary transcript expressed in skeletal muscle, N2-A (∼38% of the total sequence), and that expressed in cardiac muscle, N2-B (∼47%).

Fn3 domains are only found in the A-band region of titin, where they are arranged with Ig domains in a pattern termed super-repeats, which themselves are repeated (Fig. 1A). This region starts at the I-band / A-band junction with a unique pattern of thirteen Ig and Fn3 domains followed by the D-zone super-repeat of Ig-(Fn3)_2_-Ig-(Fn3)_3_. The seven domain D-zone super-repeat is itself repeated six times, followed by the 11-domain C-zone super-repeat of Ig-(Fn3)_2_-Ig-(Fn3)_3_-Ig-(Fn3)_3_ which is repeated 11 times (Fig. 1B). The A-band region of titin ends with a unique seven domain Ig-Fn3 pattern prior to the kinase domain at the A-band / M−band junction. Each group of titin Ig-Fn3 repeats (i.e. either Ig-Fn3, Ig-(Fn3)_2_ or Ig-(Fn3)_3_) is associated with one of the 49 layers of myosin heads in each half-thick filament, apart from the Ig-(Fn3)_6_ domain sequence near the start of A-band titin that associates with the crossbridge gap (Bennett et al., 2020).

Titin’s Fn3 domains bind myosin (Labeit et al., 1992), and the idea that the protein is closely associated with the thick filament has been confirmed in recent papers presenting the single particle cryo-electron micrographic structure of the C-zone region of isolated thick filaments (Dutta et al., 2023) and an *in situ* cryo-electron tomographic structure of the thick filament region spanning from the C-zone to the M−band (Tamborrini et al., 2023). Both structures show six titin molecules running along the thick filament in 3 loosely associated pairs, with inter-chain contacts only observed between C-zone titin Ig domains. There is currently no evidence for oligomerization of titin Fn3 domains, with short purified titin A-band fragments behaving as monomers on gel filtration (Muhle-Goll et al., 2001).

Fn3 domains have a characteristic beta sandwich fold, with a core of hydrophobic residues as the “filling” between two beta sheets that pack against each other face-to-face (Amodeo et al., 2001). Beta sheet 1 is comprised of strands A, B and E with sheet 2 formed from strands C, C’ or D,F, G1 and G2 (Muhle Goll et al., 1998). Many positions of the titin Fn3 domain sequence are either completely or highly conserved (Fig. 1D, Supplementary Fig. 2). The domain commences with a PXXP motif that is essential for correct folding (Politou et al., 1994). Strand B contains the canonical core tryptophan that is present in all titin Fn3 domains, with the only other fully conserved residue being a proline three residues C-terminal. A glycine-rich loop connects strands B and C, followed ∼ 10 residues later by a highly conserved glutamic acid on strand C that forms a salt bridge with a positively charged residue on strand F. A second tryptophan (or occasionally tyrosine) at the start of strand C’/D sits across the surface of sheet 2, and a leucine / valine in loop EF and conserved tyrosine on strand F contribute to the hydrophobic core (Hoxha and Campion, 2014). Finally, a loop of consensus NXXG connects strands F and G1 followed by a proline-rich sequence leading to strand G2 and the inter-domain linker region (Amodeo et al., 2001, Muhle Goll et al., 1998) (Fig. 1D).

Prior to this work, nine titin Fn3 domains had been structurally elucidated experimentally to high resolution: the crystal structures of Fn3-4 and Fn3-5 (also known as I110 and I111, respectively) were recently determined (Stergiou et al., 2023), Fn3-62 (A71) was determined by NMR (Muhle Goll et al., 1998), the tandems Fn3-66-Fn3-67 (A77-78) and Fn3-71-Fn3-73 (A84-86) were determined using X-ray crystallography (Bucher et al., 2010, Fleming et al., 2023) and Fn3-132 (A170) was also determined crystallographically, in the two structural contexts of its preceding two Ig domains (Mrosek et al., 2007) and succeeding kinase domain (Bogomolovas et al., 2021). Additionally, we have previously deposited a crystal structure of Fn3-3 to the protein data bank but until now have not reported any findings.

Mutations in titin have been linked to skeletal and cardiac myopathies. Dominantly inherited titin truncating mutations (TTNtv) are the most common genetic risk factor of dilated cardiomyopathy (DCM) (Herman et al., 2012) and TTNtv inherited *in trans* with either a second truncating variant or a missense variant have been identified in patients with congenital myopathies (Chauveau et al., 2014, Oates et al., 2018, Rees et al., 2021). Missense variants in Fn3-119 (A150) cause hereditary myopathy with early respiratory failure (HMERF) (Pfeffer et al., 2012), a single missense variant (Ala178Asp) in the second domain of titin (Ig-2 / Z2) has been linked to a dominantly-inherited heart disease (Hastings et al., 2016), and multiple missense, truncating and insertion / deletion variants in the final domain of titin (Ig-169 / M10) have been linked to tibial muscular dystrophy (TMD) and limb girdle muscular dystrophy type 2 J (LGMD2J) (Hackman et al., 2002).

Three titin missense variants have also been linked to hypertrophic cardiomyopathy (HCM), an inherited heart disease found in ∼ 0.2% of the population, characterized by sarcomere disorganisation and a thickening of the left ventricle wall that can lead to atrial fibrillation and cardiac arrest (Ommen et al., 2020): Arg740Leu was identified in a patient with HCM and shown to increase the affinity of titin to alpha-actinin (Satoh et al., 1999), and Arg9744His and Arg9848Gln were identified in two HCM patients with the variants reported to increase the affinity of the N2-A region of I-band titin for CARP (Arimura et al., 2009). These variants have either no or incomplete pedigree analyses, and no further supporting evidence for their pathogenicity, so might simply be rare variants of unknown significance.

High-throughput DNA sequencing has greatly increased the identification of titin variants in patients. One study (Lopes et al., 2013) of 223 HCM patients found that ∼ 61% of patients harboured rare titin missense variants, with 19% having these without co-inheriting variants in any other candidate cardiomyopathy disease genes. While some variants were enriched in the HCM cohort compared to population controls, the overall frequency of rare titin missense variants was no higher in the HCM cohort, making the pathogenicity or otherwise of these variants difficult to interpret.

Due to the size of titin, rare missense variants are not uncommon in healthy individuals, so there is a need to understand what distinguishes pathogenic from benign variants. Biochemical experiments have begun to shed light on the effect of the disease-linked missense variants on their domains: expression of Fn3-119 in bacteria show that generally dominant HMERF-linked variants render the domain insoluble, in contrast to the population variants tested (Hedberg et al., 2014). Similar experiments on the missense variants identified in congenital myopathy, heart disease and TMD/LGMD2J patients show that the variants either render the domain insoluble, prevent folding of the domain (while remaining soluble) or reduce their thermal stability by over 17° (Hastings et al., 2016, Rees et al., 2021, Rudloff et al., 2015). This contrasts with the common population variants tested, which had no destabilizing effect on their domain (Rees et al., 2021). In summary, some pathogenic titin missense variants exert their deleterious effects by reducing domain stability. Two disease hotspots, Fn3-119 and Ig-169, typically carry dominant destabilising missense variants. It remains unclear why domain destabilisation in only these two domains leads to dominant phenotypes while comparable destabilisation of other domains is associated with recessive inheritance. To support an evidence-based approach to address these questions, we pursued a biophysical approach characterising titin Fn3 domains across the titin A-band region.

Here, we present the crystal structures of seven human WT titin domains – Fn3-3, Fn3-11, Fn3-20, Fn3-49, Fn3-56, Fn3-85 and Fn3-90 – and two domains harbouring rare missense variants found in patients with HCM: Fn3-3 Arg15908His and Fn3-56 Ser23226Gly. We compare the structures and thermal stability of the domains harbouring variants to their WT counterparts, measure the effect on domain stability of three further missense variants found in patients with HCM and assess the domain environment of three variants previously linked to congenital myopathy. As mutations in A-band titin might affect any potential titin dimerisation, we also assess any evidence for domain oligomerisation.

## 2 Results

### 2.1 The titin Fn3 WT crystal structures show a conserved fold with unique structural motifs

Here we present the crystal structures of the titin domains Fn3-3, Fn3-11, Fn3-20, Fn3-49, Fn3-56, Fn3-85 and Fn3-90. All are located in the A-band region of titin, where titin associates with the thick filament. Fn3-3 and Fn3-11 are found in the non-repetitive region of A-band titin near the tip of the thick filament (Bennett and Gautel, 1996), Fn3-20 is in the second D-zone super-repeat and Fn3-49, Fn3-56, Fn3-85 and Fn3-90 are located in the 1st, 2nd, 6th and 7th C-zone super-repeats, respectively (Fig. 1A). Fn3-20 is at position 6 of the D-zone super-repeat, and Fn3-49, Fn3-56, Fn3-85 and Fn3-90 are at the 11th, 10th, 6th and 2nd position of the C-zone super-repeat, respectively (Fig. 1B). The WT structures presented in this work add to the eleven titin A-band domain structures previously determined (five Ig domains, six Fn3 domains and 1 kinase domain), and almost double the available experimentally determined titin Fn3 domain structures.

The crystal structures were determined to a resolution of between 1.05 and 1.98 Å, with 1 – 6 chains in the asymmetric unit (Table 1a and Table 1b). In general there was poor density for the first one or two N-terminal residues (corresponding to the Gly-Ser of the Gly-Ser-Ser cloning artifact) and final few C-terminal residues, but otherwise all residues could be built into the electron density maps. There was little variation in structure between chains in the asymmetric unit (where multiple copies of the domain was present), with a maximum inter-chain RMSD (C_α_, all matched residues) of 0.80 Å.

**Table 1a:** **Data collection and refinement statistics for crystal structures determined in this work.** Statistics for the highest resolution shell are shown in parentheses.

	Fn3-3 wt	Fn3-3 RH	Fn3-11 wt	Fn3-20 wt	Fn49 WT
PDB code	4o00	8OIY	8OS3	8OMW	8OSD
Wavelength (Å)	1.540	0.979	0.979	0.979	0.979
Resolution range (Å)	20.58–1.85 (1.91–1.85)	47.11–2.10 (2.18–2.10)	29.6–1.68 (1.74–1.68)	27.95–1.05 (1.09–1.05)	33.40–1.70 (1.76–1.70)
Space group	P 2_1_ 2_1_ 2_1_	P 1	C2	C2	P2_1_
Unit cell					
Dimensions (Å)	43.34 63.45 65.70	52.48 56.03 56.09	45.69 34.28 59.24	49.03 34.26 63.08	24.46 66.81 52.81
Angles (°)	90 90 90	60.06 70.16 65.62	90 92.26 90	90 99.70 90	90 102.33 90
Copies in ASU	2	6	1	1	2
Mean RMSD between chains (Å)	0.57	0.23	N/A	N/A	0.43
Total reflections	31,421 (2820)	50,185 (4925)	32,613 (2155)	669,021 (44537)	51,981 (4775)
Unique reflections	15,785 (1477)	25,780 (2523)	10,415 (943)	47,431 (4289)	17,148 (1663)
Completeness (%)	98.80 (93.90)	88.85 (86.49)	98.21 (91.20)	98.06 (89.37)	93.60 (90.53)
Mean I/sigma(I)	12.74 (1.93)	5.36 (2.35)	11.80 (3.15)	163.94 (14.17)	6.83 (1.69)
Wilson B-factor (Å ^2^)	13.47	18.87	19.62	6.98	19.41
R-pim	0.0529 (0.3663)	0.1164 (0.3238)	0.0408 (0.2518)	0.1694 (0.2372)	0.0732 (0.3858)
CC1/2	0.997 (0.699)	0.973 (0.719)	0.993 (0.893)	0.782 (0.602)	0.989 (0.77)
Reflections used in refinement	15,784 (1477)	25,777 (2523)	10,414 (943)	47,429 (4288)	17,143 (1663)
Reflections used for R-free	788 (75)	1367 (130)	567 (55)	2010 (183)	900 (96)
R-work	0.1679 (0.2317)	0.2061 (0.2323)	0.1658 (0.2410)	0.1489 (0.1634)	0.2171 (0.3166)
R-free	0.2093 (0.2571)	0.2565 (0.2805)	0.2081 (0.2911)	0.1772 (0.2009)	0.2603 (0.3164)
Number of non-hydrogen atoms	1946	4776	917	1114	1714
macromolecules	1611	4514	794	853	1631
ligands	0	0	0	1	0
solvent	335	262	123	260	83
Protein residues	206	606	103	107	206
RMS (bonds) (Å)	0.003	0.002	0.010	0.010	0.007
RMS (angles) (°)	0.84	0.51	1.19	1.27	0.90
Ramachandran favored (%)	96.04	99.16	97.03	99.05	97.03
Ramachandran outliers (%)	0	0	0	0	0
Clashscore	0.94	0.46	6.23	0.58	1.26
Average B-factor (Å^2^)	14.37	24.54	26.80	12.65	27.06
Macromolecules (Å^2^)	12.49	24.59	25.85	9.22	26.75
Ligands (Å^2^)	N/A	N/A	N/A	9.14	N/A
Solvent (Å^2^)	23.40	23.58	32.95	23.91	33.17

**Table 1b:** Data collection and refinement statistics for crystal structures determined in this work. Statistics for the highest resolution shell are shown in parentheses. Mean RMSD between chains in asymmetric unit (ASU) calculated using US-Align (C_α_, all residues) (Zhang et al., 2022). Ligands in the structures are Cl^-^ (Fn3-20 wt and Fn3-56 wt), Zn^2+^ and Cl^-^ (Fn3-56 SG) and Na^+^ and Cl^-^ (Fn3-85 wt).

	Fn3-56 wt	Fn3-56 SG	Fn3-85 wt	Fn3-90 wt
PDB code	8OQ9	8ORL	8OT5	8OTY
Wavelength (Å)	0.979	0.979	0.969	0.979
Resolution range (Å)	36.52–1.65 (1.71–1.65)	49.15–1.43 (1.48–1.43)	57.19–1.56 (1.62–1.56)	28.32–1.90 (1.97–1.90)
Space group	C 2 2 2_1_	C 2 2 2_1_	P2_1_	P2_1_
Unit cell				
Dimensions (Å)	70.53 89.63 97.11	70.35 89.86 98.31	59.36 99.83 62.24	25.08 137.46 51.55
Angles (°)	90 90 90	90 90 90	90 105.55 90	90 104.02 90
Copies in ASU	3	3	6	4
Mean RMSD between chains (Å)	0.61	0.27	0.40	0.40
Total reflections	145,415 (6916)	373,628 (28837)	336,839 (31140)	70,240 (4978)
Unique reflections	35,649 (2622)	57,214 (5192)	98,482 (9760)	25,557 (2190)
Completeness (%)	95.44 (71.37)	99.09 (91.26)	99.30 (98.83)	96.88 (84.68)
Mean I/sigma(I)	17.16 (2.20)	11.25 (1.89)	10.29 (2.79)	6.15 (1.83)
Wilson B-factor (Å^2^)	16.80	15.09	18.15	21.58
R-pim	0.0303 (0.2558)	0.0412 (0.3223)	0.0572 (0.2366)	0.0618 (0.3093)
CC1/2	0.999 (0.765)	0.995 (0.703)	0.988 (0.831)	0.992 (0.778)
Reflections used in refinement	35,649 (2622)	57,213 (5192)	98,455 (9755)	25,548 (2189)
Reflections used for R-free	1791 (129)	2870 (261)	4903 (492)	2411 (206)
R-work	0.1670 (0.2824)	0.1586 (0.2401)	0.1700 (0.2349)	0.2033 (0.3047)
R-free	0.1939 (0.2925)	0.1941 (0.2973)	0.1966 (0.2531)	0.2494 (0.3413)
Number of non-hydrogen atoms	2711	2800	5935	3434
macromolecules	2333	2346	4985	3157
ligands	8	10	5	0
solvent	370	444	945	277
Protein residues	305	305	621	403
RMS(bonds) (Å)	0.011	0.097	0.008	0.006
RMS(angles) (°)	1.09	0.99	1.00	0.63
Ramachandran favored (%)	97.66	97.99	97.70	97.47
Ramachandran outliers (%)	0	0	0	0
Clashscore	2.59	1.28	2.90	0.32
Average B-factor (Å^2^)	21.51	20.88	24.35	25.36
Macromolecules (Å^2^)	20.43	19.01	22.54	25.20
Ligands (Å^2^)	28.02	23.82	25.92	N/A
Solvent (Å^2^)	28.18	30.68	33.91	27.25

All seven structures present a typical Fn3 fold of a beta sandwich composed of two beta sheets encompassing a hydrophobic core. Alignment of their primary sequences show an insert in Fn3-20 extending the loop connecting strands C'/D to E by five residues that does not align with residues in any other titin Fn3 domain (Fig. 1D, Supplementary Fig. 1). That aside, the primary sequences are highly homologous with the domains structurally determined in this work aligning with identical residues at 14 positions.

The extended loop in Fn3-20 results in a unique protrusion from the back “side” of the domain, not present in the other structures presented here, nor any other titin Fn3 domain structurally determined.

### 2.2 Titin Fn3-3 and Fn3-56 domains harbouring rare missense variants in HCM patients differ little in structure from WT

In addition to the WT domains, the structures of Fn3-3 Arg15908His and Fn3-56 Ser23226Gly were determined to a resolution of 2.10 and 1.43 Å, respectively. The two rare missense variants have each been identified in 12 patients of an 874-patient HCM cohort at UCLH (Lopes et al., 2013), with general population minor allele frequencies (MAFs) of 6.5 × 10^-3^ and 7.2 × 10^-3^ for Arg15908His and Ser23226Gly, respectively, suggesting they may play a role in the patients’ disease. There is usually a positively charged residue at the Arg15908 domain position, and the Ser23226 position is also highly conserved (Fig. 1D, Supplementary Fig. 1). As missense variants in titin have previously been linked to myopathies (Rees et al., 2021), including cardiomyopathies (Arimura et al., 2009, Satoh et al., 1999), a crystal structure of the variant domain may help elucidate a molecular pathomechanism.

**Fig. 2:**
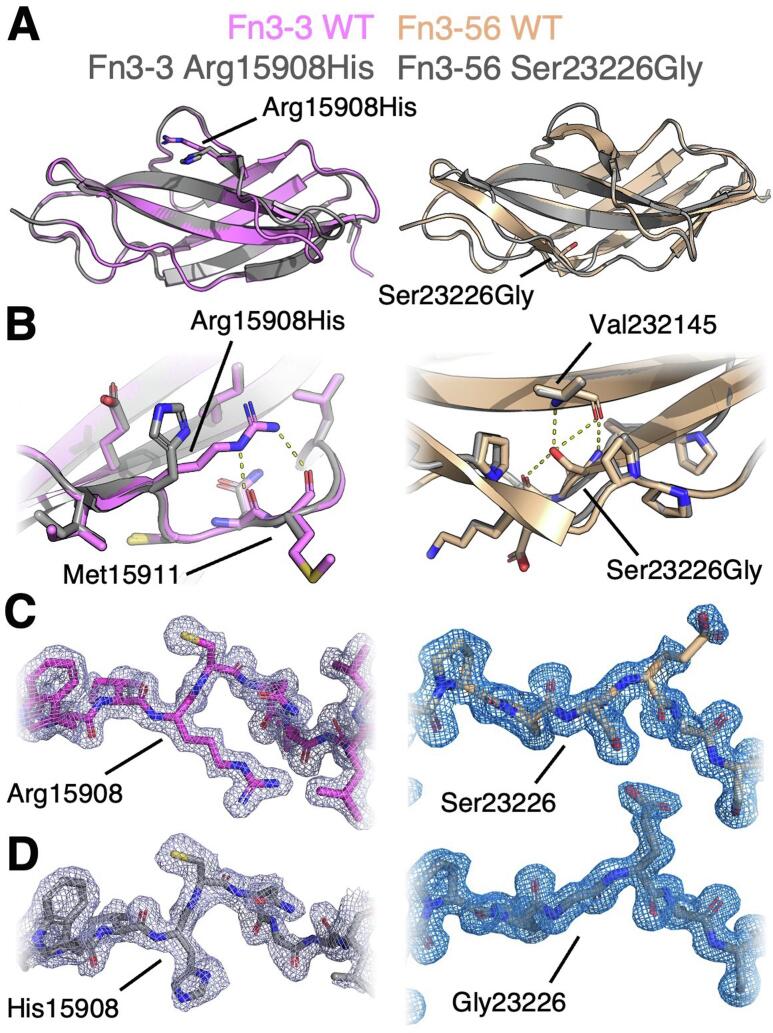
**Comparison of WT and variant Fn3-3 (left) and Fn3-56 (right) domain crystal structures. A:** cartoon representation of aligned WT and variant domain structures, with mutated residue sidechains shown in stick representation. **B:** detail of region surrounding mutated residue. Sidechains shown in stick representation. **C and D:** detail of electron density surrounding WT (C) residue mutated in variant structure (D). 2Fo-Fc maps displayed at sigma = 1.0.

Both variant structures differed little compared to their WT counterparts (Fig. 2), with a maximum RMSD between one WT and variant chain of 0.70 Å for Fn3-3 and 0.61 Å for Fn3-56. The sidechain of Arg15908 in Fn3-3 wt (Arg54 in PDB 4O00) forms H-bonds with the main chain carboxyl groups of Asn15910 (Asn56) and Met15911 (Met57), which are lost in the Arg15908His variant structure. In Fn3-56, the hydroxyl group of the Ser23226 (Ser92 in PDB 8OQ9) sidechain adopts an intriguing conformation where, instead of pointing out into the solvent, it faces inwards and forms a hydrogen bond with the mainchain of Val23214 (Val80) on strand F as well as Glu23227 (Glu93); these polar contacts are lost in the Ser23226Gly structure.

Analysis of the dynamics of ^15^N-labelled Fn3-56 using 2D NMR showed that the mutation of Ser23226 to glycine increased the flexibility of the variant domain; interestingly, ^15^N relaxation (Supplementary Fig. 3A-C) and solvent exchange (Supplementary Fig. 3D) experiments suggest that this increased flexibility does not occur on fast ps-ms timescales but on a much slower ms-s timescale (with increased solvent exchange for Fn3-56 Ser23226Gly even at residues with low accessible surface area, Supplementary Fig. 3E), indicating a robust integration of the strand harbouring the mutated residue into the correctly folded domain.

### 2.3 Tested rare titin missense variants from HCM patients affect the stability or folding of their domain

Missense variants in titin domains linked to myopathies have previously been shown to reduce the thermal stability of the domain by at least ∼ 17 °C (Rees et al., 2021), prevent soluble expression of the protein when expressed in E. coli (Hedberg et al., 2014), and/or resulted in a soluble but unfolded protein (Rudloff et al., 2015). We therefore characterised the effect of the variants Arg15908His and Ser23226Gly on their domain, along with three further rare missense variants: Ala16872Pro, Ser27083Pro and Arg27839Gln. Ala16872Pro (population MAF 7.2 × 10^-6^) was identified in a patient in the UCLH HCM cohort co-inherited with the variant Glu259* in *PKP2* (population MAF 4.0 × 10^-6^), the gene encoding the cardiac desmosomal protein plakophilin; Arg27839Gln (population MAF 4.3 × 10^-4^) was identified in a patient in the same cohort co-inherited with two further rare titin missense variants (Thr24311Ala and, Ile30525Val), but their higher population allele frequencies (4.5 × 10^-3^ and 6.5 × 10^-3^, respectively) and unsuspicious residue exchanges led us to not pursue their study; finally, Ser27083Pro (population MAF 2.6 × 10^-4^) was identified in one patient (of ∼ 15 genotyped) in the Guy’s and St Thomas’ Trust HCM cohort, co-inherited with the Lys60Asn variant (population MAF 4.0 × 10^-3^) in the cardiac actin-binding protein nebulette (encoded by *NEBL*).

**Fig. 3:**
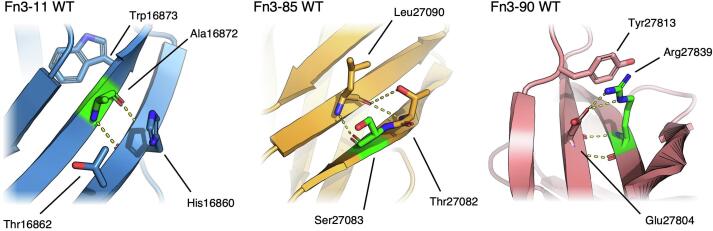
**Position of residues in crystal structures of Titin Fn3-11, Fn3-85 and Fn3-90 mutated in patients with hypertrophic cardiomyopathy.** Ala16872Pro, Ser27083Pro and Arg27839Gln were identified in patients from HCM cohorts. Residue mutated in patients highlighted in green, polar contacts shown as dashed yellow lines. Residues of interest shown in stick representation.

Ala16872 (Ala25 in PDB 8OS3) is located on strand B in Fn3-11, one residue N-terminal of the core, conserved tryptophan. This position is not conserved, with proline, tyrosine and phenylalanine being the only residues absent from this position in any titin Fn3 domain. Ala16872 forms 2 hydrogen bonds with His16860 (His13) on strand A as part of sheet 1 (Fig. 3); mutation to the “secondary structure breaker” proline would result in a loss of the bond linking the mainchain amine of the mutated residue to the mainchain carbonyl of His16860.

Ser27083 (Ser16 in PDB 8OT5) is on strand A in Fn3-85 and whilst not a conserved position, proline is never located here (Fig. 3). The residue forms 1 hydrogen bond via its carbonyl group with Leu27090 (Leu23) on strand B as part of sheet 1; due a bulge in strand A, Leu27090 forms its second mainchain beta sheet hydrogen bond with Thr27082 (Thr15). Mutation of Ser27083 to proline may result in minor steric clashes with Leu27090 and Thr27082 but would not alter the hydrogen bond network seen in the WT structure.

Arg27839 (Arg78 in PDB 8OTY) is on strand F of Fn3-90 and is at the highly conserved position where in 125 of the 130 titin Fn3 domains a positively charged residue is predicted to form a salt bridge with an aspartic or glutamic acid on strand C. In Fn3-90, Arg27839 forms 2 main chain hydrogen bonds with Glu27804 (Glu43) on strand C, and a salt bridge via their sidechains (Fig. 3). The epsilon nitrogen of the arginine sidechain is also positioned to donate a hydrogen bond to the phenol ring on Tyr27813 (Tyr52). Mutation of Arg27839 to glutamine would result in the loss of the salt bridge with Asp27804 but may still be able to form hydrogen bonds with Asp27804 and Tyr27813, depending on its conformation. Structural predictions of the three variant domains suggest little change to the backbone trace of the proteins and that no clashes would be introduced upon mutation (Supplementary Fig. 4, Supplementary Table 5).

Bioinformatic predictions on the effects of the variants (Pires et al., 2014) predict all five variants to be destabilising, but predictors of the deleterious or disease-causing effects of each variant (Adzhubei et al., 2013, Ng and Henikoff, 2001, Rentzsch et al., 2019) result in contradictory conclusions (Supplementary Table 4) which in general do not correlate with each other (Supplementary Fig. 2). Calculation of the change in Gibbs free energy due to the mutations using FoldX suggests either no change compared to WT (Fn3-56 Ser23226Gly and Fn3-90 Arg27839Gln) or that the mutation is destabilising (Fn3-3 Arg15908His, Fn3-11 Ala16872Pro and Fn3-85 Ser2708Pro) (Supplementary Table 5).

**Fig. 4:**
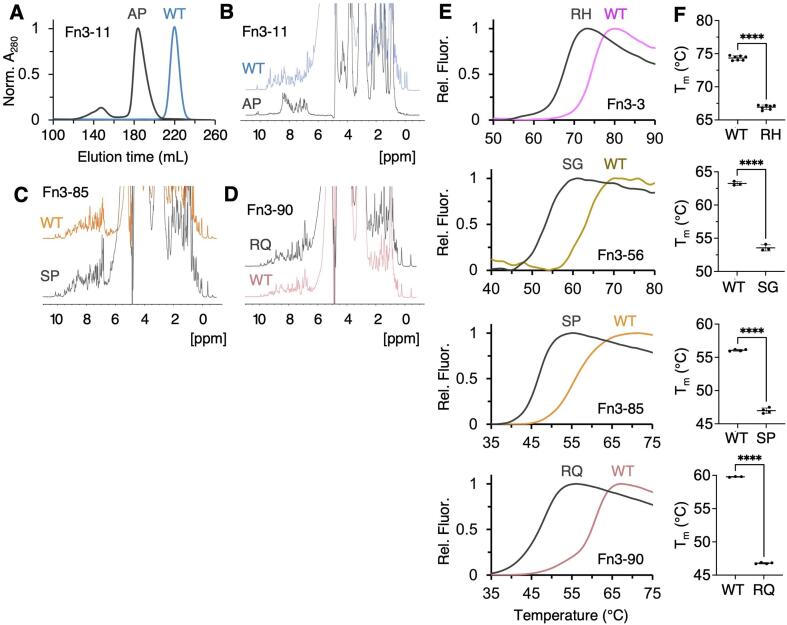
**Biophysical analysis of purified titin domains with variants from HCM patients. A:** analytical gel filtration traces of Fn3-11 wt and Ala16872Pro. **B:** 1D NMR of Fn3-11 wt and Ala16872Pro. **C:** 1D NMR of Fn3-85 wt and Ser27083Pro. **D:** 1D NMR of Fn3-90 wt and Arg27839Gln. **E:** differential scanning fluorimetry (DSF) plots showing unfolding of WT Fn3-3, Fn3-56, Fn3-85 and Fn3-90 and their HCM-linked variants Arg15908His, Ser23226Gly, Ser27083Pro and Arg27839Gln, respectively. **F:** Melting temperatures, T_m_, of each domain calculated from DSF unfolding curves. Statistical difference between the means of WT and variant melting temperatures calculated using the student’s *t*-test. *n* = 3–5 technical repeats, **** = *P* < 0.0001.

All domains with variants from HCM patients were solubly expressed in bacteria (data not shown), however Fn3-11 Ala16872Pro was unfolded as evidenced by 1D NMR (Fig. 4B) and its decrease in elution volume (and increased hydrodynamic radius) compared to WT in size exclusion chromatography (Fig. 4A). The other four variants were folded (Fig. 2, Fig. 4C-D) so we compared the thermal stability of these WT and variant domains using differential scanning fluorimetry. The melting temperatures, T_m_, are shown in Table 2; briefly, the WT domains had T_m_s of between 59.8 and 74.3 °C and the variant domains had T_m_s between 46.8 and 66.9 °C, with a reduction in thermal stability ranging from 7.4 °C for Fn3-3 Arg15908His to 13.0 °C for Fn3-90 Arg27839Gln.

**Table 2:** Melting temperatures (T_m_) of titin Fn3-3, Fn3-56, Fn3-85, Fn3-90 and their variants from HCM patients.

		Melting temperature (℃)		
Domain	Variant	WT	Variant	Delta (℃)
Fn3-3	Arg15908His	74.3 ± 0.3	66.9 ± 0.3	7.4
Fn3-56	Ser23226Gly	63.2 ± 0.3	53.4 ± 0.5	9.8
Fn3-85	Ser27083Pro	56.1 ± 0.1	47.0 ± 0.4	9.1
Fn3-90	Arg27839Gln	59.8 ± 0.1	46.8 ± 0.1	13.0

### 2.4 Variant residues in patients with congenital myopathy

Three of the domains presented here harbour missense variants linked to early onset myopathy, a muscle disease of heterogeneous symptoms that typically presents at a young age and can involve heart disease depending on whether the variants are expressed in the predominant titin transcript, N2B, that is expressed in the heart. The inheritance pattern of titin variants linked to early onset myopathy is typically compound heterozygous, where one variant is inherited from the mother and a second variant, also in titin, is inherited from the father. Most patients so far identified have two truncating / splice site variants or one truncating and one missense variant inherited *in trans*, while homozygous missense variants are rare. The parents do not suffer from the disease, although several families have a history of heart disease of various types, consistent with the observation that truncating TTN variants are a major risk factor for dilated cardiomyopathy in heterozygosity but with incomplete penetrance (Schafer et al., 2017).

The variant in Fn3-20, Leu18237Pro, was identified in a patient also carrying two C-terminal truncating variants inherited *in trans*, whilst Val22232Glu in Fn3-49 was co-inherited *in trans* with a truncating variant in the kinase domain at the A-band / M−band junction. Gly27849Val in Fn3-90 was co-inherited *in trans* with an A-band truncating variant. All three patients (identified as patients 29, 22 and 24 respectively in (Rees et al., 2021)) had at least two TTN variants expressed in the N2B transcript and accordingly all three suffered from heart disease, with patient 22 requiring a heart transplant at 14 years of age ((Chauveau et al., 2014), identified as patient 4).

Fn3-20 Leu18237Pro was solubly expressed but caused a 16.9 °C reduction in domain thermal stability. Conversely, Fn3-49 Val22232Glu and Fn3-90 Gly27849Val were insolubly expressed in bacteria and following attempts to refold the protein after purification from the insoluble fraction, were shown to be unfolded by 1D NMR (Rees et al., 2021).

**Fig. 5:**
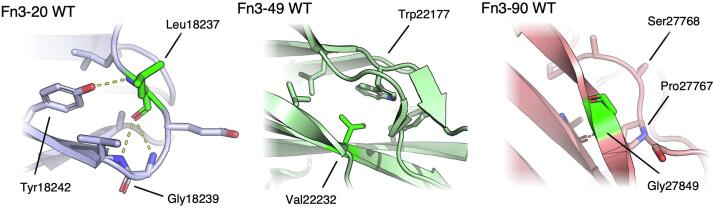
**Position of residues in crystal structures of Titin Fn3-20, Fn3-49 and Fn3-90 mutated in congenital myopathy patients.** Leucine 18,237 mutated to proline in Fn3-20, valine 22,232 mutated to glutamic acid in Fn3-49 and glycine 27,849 mutated to valine in Fn3-90 in patients with congenital myopathy, co-inherited with either one truncating variant in trans (Fn3-49 and Fn3-90) or two truncating variants (Fn3-20). Residue mutated in patients highlighted in green, hydrogen bonds shown as dashed yellow lines. Residues of interest shown in stick representation.

The crystal structures of the WT Fn3-20, Fn3-49 and Fn3-90 domains help rationalize these observations (Fig. 5). Leu18237 (Leu74 in PDB 8OMW) is located on a loop connecting strands E and F in Fn3-20, is most commonly leucine and is only a proline in 1 titin Fn3 domain (Fn3-126). Mutation to proline would abolish the hydrogen bond from the leucine main chain amine group to the side chain hydroxyl group of Tyr18242 (Tyr79), reducing the stability of the domain.

Val22232 (Val81 in PBD 8OSD) is on strand F, is largely conserved in titin Fn3 domains and contributes to the hydrophobic core, with the side chain making a large contact with the canonical tryptophan. Mutation to glutamic acid would introduce a charged sidechain into the core of the domain, which likely renders correct folding of the domain impossible.

Gly27849 (Gly88 in PDB 8OTY) in Fn3-90 is located at a highly conserved position post-FG loop. Mutation to valine at this buried position introduces a large sidechain facing toward the N-terminal PXXP motif crucial for correct Fn3 domain folding (Politou et al., 1994). The disruption of this motif is likely what renders Fn3-90 Gly27849Val insoluble when expressed in bacteria, and unable to be refolded.

The predicted structures of the three variant domains suggest they can in principle fold correctly, with a slight clash between Val27849 and Ser27766 in Fn3-90 Gly27849Val the only steric hindrance observed, but calculation of the change in Gibbs free energy due to the mutations suggests all three destabilise their domain (Supplementary Fig. 4, Supplementary Table 5).

### 2.5 Analysis of dimer interfaces show no evidence for physiological dimerization

Six titin filaments run along each thick filament in the A-band region of the sarcomere. Recent cryo-electron micrographic and cryo-electron tomographic structures show no evidence that titin Fn3 domains dimerise in the assembled C-zone region of the thick filament (Dutta et al., 2023, Tamborrini et al., 2023), but the conformation of titin in the D-zone and ends of the thick filament are yet to be elucidated. We therefore studied the contacts between molecules in each crystal structure to identify any dimerization interfaces that could be physiologically relevant, for example during assembly intermediates of the thick filament. We made two assumptions to consider if the interface was physiologically relevant: first, that the interface with the greatest interacting area would be the most likely physiological interaction and secondly that both chains of the molecule should be facing the same direction with the interaction involving the sides (rather than the N- or C-terminal “ends”) of the domain. Additionally, if titin twisted around each other to form a helix, the likely interaction would be based on a two-fold rotational symmetry (around the N- to C-axis) and so the residues involved in the interaction would be identical for each chain.

All domains eluted from size-exclusion chromatography as a monomer (data not shown), but the inter-chain contacts in the crystal structures could reveal physiological interfaces that would mediate dimerization in the full-length protein.

**Fig. 6:**
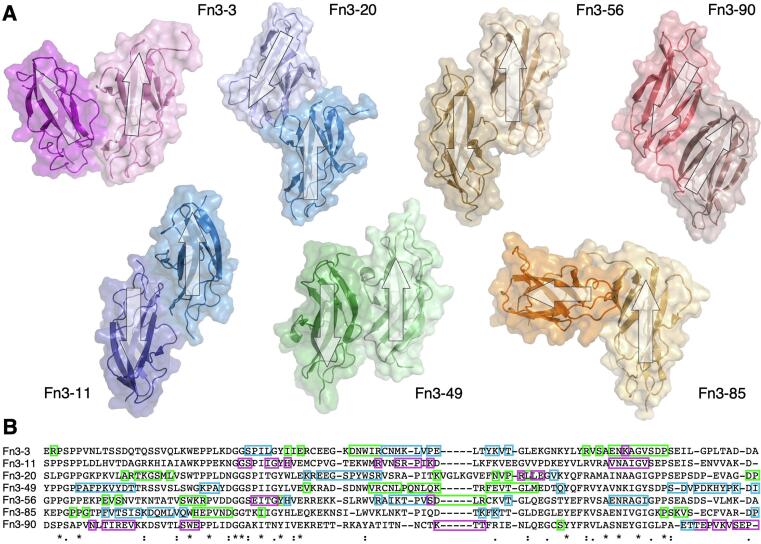
**Assessment of domain-domain interactions within crystal. A:** For each determined WT crystal structure, the homo-dimeric interaction with the largest surface area is presented, with the relative orientation of each protein chain indicated by the arrow pointing towards the C-terminus. **B:** alignment of the domain sequences, with the residues involved in the interacting surface in (arbitrary) chain A boxed in green, chain B boxed in blue and both chains boxed in purple.

Fig. 6A shows two chains of each WT structure with the greatest interacting area as assessed using PDBePISA (Krissinel and Henrick, 2007). Of the seven domains, only Fn3-3 presents the two chains facing the same direction, with the interaction involving residues on the sides of the domain. Each chain has a different interaction interface, with only one shared interface residue, with the arginine mutated to histidine in the HCM cohort forming part of one interface (Fig. 6B). Despite a Δ^i^G P-value of 0.19 calculated by PDBePISA (suggesting this interface results in a greater drop in free energy, Δ^i^G, than an interface composed of random surface residues), the interface had a complexion significance score (CSS) of 0, suggesting the interface is not physiologically relevant.

As well as interfaces with the greatest interacting area, those that had a non-zero CSS and a physiologically relevant orientation were also investigated. However, this only identified an interaction in the Fn3-85 crystal structure where two cysteines at the C-terminus of the domain – residues 27,164 (Cys97) and 27,171 (Cys104) – form two disulphide bridges with the chains related by two-fold rotational symmetry around the axis running parallel to the domains. We consider this interaction highly unlikely to be physiological as the second cysteine forms part of the PXXP loop at the start of the following domain (Fn3-86) and is predicted to be buried in vivo.

## 3 Discussion

Here we present the crystal structures of seven WT and two variant Fn3 domains from across the A-band region of the giant sarcomeric protein titin. The structures increase the coverage of titin’s primary sequence with experimentally determined high resolution structural information by ∼ 15% and nearly double the coverage of A-band titin, with the first experimental structure from the D-zone (Fn3-20). We use these to assess the impact of disease-linked missense variants and test for evidence of titin oligomerization.

All domains determined present a typical Fn3 domain fold, with the extended loop connecting strands C’/D and E in Fn3-20 being the largest difference between the structures. With this extended loop only present in one further domain (Fn3-25), this could be a candidate for a (semi-) unique binding site on a titin Fn3 domain for a putative ligand, analogous to the “KTLE loop” in Ig-159 (A169) required for MuRF1 binding to titin (Bogomolovas et al., 2014). The two variant domains, which each harbour a rare missense variant found in a cohort of HCM patients, closely align with their respective WT domains, with the mutated residue incorporated into the domain without affecting its conformation. Both the Arg15908His variant in Fn3-3, and Ser23226Gly variant in Fn3-56, however, result in the loss of polar contacts, and this is reflected in the loss of thermal stability of the domains. We also characterised the effects of three further rare missense variants found in patients with HCM: Fn3-11 Ala16872Pro renders its domain unfolded (contrary to the predicted domain structure) but is still solubly expressed in bacteria, whilst Fn3-85 Ser27083Pro and Fn3-90 Arg27839Gln do not affect the folding of their domain but reduce its thermal stability.

We and others have previously shown that many titin missense variants linked to disease reduce the thermal stability of their domain by ∼ 17 °C or result in insoluble and/or unfolded protein (Hedberg et al., 2014, Rees et al., 2021, Rudloff et al., 2015). The folded Fn3-3 domains with variants from HCM patients studied here reduce thermal stability by 7–13 °C. This is between the values we see for both common variants (Rees et al., 2021) and variants with debatable links to pathogenicity (Fukuzawa et al., 2021), and *bona fide* disease-linked variants. Either these variants do not contribute to disease or they reflect the late onset and incomplete penetration of the disease profile of HCM. The high melting temperatures for two of the variant domains (66.9 °C for Fn3-3 Arg15908His and 53.4 °C for Fn3-56 Ser23226Gly) suggest that for these it is the former, as previously characterized variants linked to disease via domain instability typically reduce the domain melting temperature to below physiological levels. Although Fn3-85 Ser27083Pro and Fn3-90 Arg27839Gln have melting temperatures above physiological temperatures (47.0 °C and 46.8 °C, respectively) their unfolding curves show a proportion of protein unfolds below 37 °C, which may point to these mutations playing a role in disease. The various interactions experienced by these individual domains within muscle may however effect their thermal stability such that some domains have higher *in vivo* stability, and the directional mechanical force that may be experienced by titin during the muscle contraction cycle could also differentially affect variants, complicating the ability to draw conclusions from isolated thermal stability measurements.

For a clearer picture to emerge, further measurements of the melting temperatures of proteins/domains with disease-linked and neutral variants will have to be undertaken to establish if a clear cut-off value for destabilization and/or absolute melting temperature can be discovered that distinguishes neutral variants from those that cause disease via their misfolding or destabilisation. The large variance in T_m_ values for the WT domains measured in this study could suggest “hotspot” domains (with low WT T_m_ values) that are more likely to be affected by mutation, although the limited data available for disease-linked missense variants in titin does not currently provide evidence for this hypothesis.

The negative effects of Ala16872Pro on Fn3-11 are easier to rationalise as it renders the domain unfolded, though soluble. For two variants identified in congenital myopathy patients, we have previously shown that missense variants that render the domain misfolded and insoluble when expressed in bacteria also do the same when expressed in cardiac and skeletal cells (Rees et al., 2021), giving no reason to suggest expressing Fn3-11Ala16872Pro in mammalian cells would “rescue” folding of the domain via chaperone-mediated folding. Increasing the hydrodynamic radius of Fn3-11 due to unfolding would leave the full-length titin molecule out of register for binding to its partners on the thick filament and could affect its function as a molecular ruler and putative role in length-dependent activation, but could also result in the recruitment of components of the proteostasis systems and cause an “overload” of these, or introduce non-native interactions with other sarcomeric components, which has previously been observed in or hypothesised for other disease-linked missense variants in titin (Bogomolovas et al., 2016, Jiang et al., 2021, Rees et al., 2021).

Complementary to the efforts to classify destabilising missense variants identified in folded titin domains by biophysical measurements would be larger pedigree analysis, as the variants studied here are from large patient cohorts without information regarding the genetic and phenotypic status of probands’ relatives.

Titin was predicted to be a dimer along its A-band region (Al-Khayat et al., 2013), and has recently been shown to loosely associate in pairs along the C-zone region of the A-band, however with no dimerization between Fn3 domains (Dutta et al., 2023, Tamborrini et al., 2023). We see no evidence for dimerization in the crystal structures of the C-zone Fn3 domains determined here (Fn3-49, Fn3-56, Fn3-85 and Fn3-90), agreeing with these findings, and our crystal structures of Fn3 domains in the D-zone and at the end of the thick filament suggest the same organisation of titin Fn3 domains will also be found here. Further structural work, particularly of titin in its native environment, will hopefully answer these questions and help in the quest to distinguish pathogenic from neutral missense variants.

## 4 Materials and methods

### 4.1 Cloning

DNA sequences encoding the wild type (WT) titin domains Fn3-3, Fn3-11, Fn3-20, Fn3-49, Fn3-56, Fn3-85 and Fn3-90 were amplified from a human skeletal muscle cDNA library and cloned into a vector modified from pET14b (Millipore) containing an N-terminal His_6_ tag followed by a TEV cleavage sequence. The vectors encoding domains Fn3-3 Arg15908His, Fn3-11 Ala16872Pro, Fn3-56 Ser23226Gly, Fn3-85 Ser27083Pro and Fn3-90 Arg27839Gln (variants numbered according to the inferred complete, IC, sequence NM_001267550) were generated by point mutagenesis of the WT vector. For domain boundaries, see Supplementary Table 1.

### 4.2 Protein expression and purification

Modified pET vectors encoding the titin domains were transformed into *E. coli* BL21 (DE3)-RIPL (Agilent) using the heat shock method and cultured overnight in 5–20 mL LB broth with 50 μg/mL ampicillin, 25 μg/mL chloramphenicol at 37 °C with shaking at 250–300RPM. 0.5–1L LB or Terrific broth with ampicillin was inoculated with overnight culture at a 1:50 dilution and grown at 37 °C with shaking to an optical density (OD_600_) of 0.6–0.8. Protein expression was induced with 0.1–0.5 mM IPTG and the cultures grown at 18 °C overnight with shaking, then harvested by centrifugation at 4000g, resuspended in buffer A (30 mM Tris pH 7.5, 300 mM NaCl, 30 mM imidazole, 14 mM beta-mercaptoethanol) and flash-frozen in liquid nitrogen. After thawing, cells were lysed and DNA digested by incubation with 0.1 mg/ml lysozyme and 10 μg/ml DNase I on ice for 30′, followed by sonication for 5′. The soluble and insoluble fractions were separated by centrifugation at 30,000g for 40 mins, and after passing through a 5 μm filter the soluble fraction was loaded onto a HisTrap column (GE) equilibrated with buffer A. Following washing with 10 column volumes buffer A, the his-tagged protein was eluted with 3–5 column volumes of buffer B (30 mM Tris pH 7.5, 300 mM NaCl, 500 mM imidazole, 14 mM beta-mercaptoethanol) and promptly exchanged back into buffer A by passing through a PD-10 column equilibrated in buffer A. The His_6_ tag was then cleaved by incubation with 50μg/ml TEV protease for ∼ 10 h and the protein was separated from uncut protein and cleaved tag by passing through a second HisTrap column. The flow-through was injected onto a Superdex75 (GE) equilibrated in either 20 mM HEPES or 20 mM Tris pH 7.5 with 100 mM NaCl and 1 mM DTT, collected fractions were run on SDS-PAGE and those containing pure protein were collated, concentrated, flash frozen and stored at −80 °C for later use.

### 4.3 Crystallization

Purified protein was concentrated to 10–45 mg/ml and MRC sitting drop 96-well crystallization plates were setup with the JSCG+, PACT, and Nextal Classics I & II screens (100ul per well), using a Mosquito (SPT LabTech) to mix 0.2ul protein and 0.2ul reservoir solution in the sample subwells. Conditions in which protein crystals grew were optimized if necessary by screening around the precipitant concentration and buffer pH in the initial hit. Prior to data collection crystals were flash frozen in liquid nitrogen with no additional cryobuffers. For the crystallization conditions used to grow a crystal of each titin domain, see Supplementary Table 2.

### 4.4 X-ray data collection

All data was collected at Diamond Light Source (UK) except for Fn3-3 wt, which was collected on an Xcalibur (Oxford Diffraction) X-ray source. In general, 180° of data was collected at 0.1° rotation per frame at 100 K and a single crystal was used for each structure determination. For data collection parameters for each crystal, see Supplementary Table 3.

### 4.5 Structure solution and refinement

Indexing and integration of diffraction data was performed either in iMOSFLM (Powell et al., 2017) or using the automatic processing suite at Diamond Light Source, with scaling and merging then performed using AIMLESS (CCP4) (Winn et al., 2011). Molecular replacement was run in PHASER (McCoy et al., 2007): for Fn3-3 wt, residues 99–197 of PDB 3LPW (Bucher et al., 2010) was used as the search model; for all other structures, Fn3-3 wt was employed. The molecular replacement solution was rebuilt and refined in PHENIX.REFINE (Liebschner et al., 2019) and COOT (Emsley et al., 2010). Simulated annealing was employed for the first round of refinement, with XYZ and B-factor refinement employed in the first and all subsequent rounds. For refinement of Fn3-20 wt and Fn3-56 Ser23226Gly, anisotropic B-factor refinement of all non-hydrogen protein atoms was employed; for all other structures a combination of individual atom B-factor refinement and TLS group refinement was used. Non-crystallographic symmetry restraints were not employed during refinement. Secondary structures were assigned to the models using STRIDE (Frishman and Argos, 1995) in 2StrucCompare (Drew and Janes, 2019). For further details and refinement statistics see Table 1a and Table 1b.

### 4.6 Differential scanning fluorimetry

Purified protein and SYPRO orange (Life Technologies) were mixed to a final concentration of 20 μM and 20X, respectively, and 20 μl per sample were pipetted into a well of a 96-well PCR plate (BioRad). The plate was loaded into an mx3005p qPCR machine (Stratagene) and the program “_RunProtocol_SyproOrange_25to95C-1KperMin_Mx3005p.mxp” retrieved from ftp://ftp.sgc.ox.ac.uk/pub/biophysics (Niesen et al., 2007) was run (temperature ramp from 25 to 95 °C at a rate of 1 °C/min with excitation at 492 nm (FAM filter) and emission recorded at 610 nm (ROX filter). The data was exported to excel with the region of interest determined automatically by the spreadsheet “DSF Analysis v3.0.2.xlsx” and the melting temperature, T_m_, was calculated in Prism 8.0 (GraphPad) by fitting to the Boltzman equation. The statistical differences between the means of the technical repeats were calculated using the student’s *t*-test and plotted in Prism 8.0.

### 4.7 Nuclear magnetic resonance

The 1D 1H spectra of WT and variant Fn3-11, Fn3-85 and Fn3-90 was collected as in (Rees et al., 2021). ^15^N labelled Fn3-56 wt and Ser23226Gly were produced as described previously (Rostkova et al., 2015). All NMR data presented here were recorded on a Bruker Avance III 500 MHz spectrometer fitted with a cryoprobe at a protein concentration of 0.5 mM in 20 mM sodium phosphate buffer (pH 7.2) with 50 mM sodium chloride, 2 mM DTT and 0.02 % NaN3 at a temperature of 298 K.

Backbone H_N_ and proton resonances for the WT and Ser23226Gly mutant domain were assigned using a combination of ^15^N resolved 3D NOESY-HSQC and TOCSY-HSQC. Experiments were recorded with standard pulse sequences for watergate-supported (Piotto et al., 1992) versions of the experiments provided by the manufacturer.

^15^N relaxation was measured using standard T_1_, T_2_ and heteronuclear NOE experiments provided by the manufacturer. All experiments are based on a standard ^1^H-^15^N HSQC experiment with watergate for water suppression and recorded as a pseudo 3D experiment. Values are provided only for residues that do not show substantial peak overlap in the 2D HSQC.

Water exchange was quantified by analysis of water-H_N_ exchange crosspeak heights observed at 4.78 ppm in 3D NOESY-HSQC spectra recorded with a mixing time of 100 ms and normalised by dividing by the peak height of the corresponding diagonal peak. Only residues with no overlap in the 2D HSQC could be analysed accurately. In addition, some amino acids have α-protons at the same chemical shift as the water so that the analysis could not be carried out in those cases.

### 4.8 Alignments

The primary sequences of all titin Fn3 domains were aligned using Clustal Omega (Sievers et al., 2011) using the default parameters. A structural alignment of the crystal structures determined was generated with PDBeFold (Krissinel and Henrick, 2004) using default parameters. Pairwise RMSDs (C_α_, all matched residues) were calculated in US-Align (Zhang et al., 2022).

### 4.9 Interface analysis

The dimer interface within each crystal structure with the largest area was identified by loading the PDB file into EBI Pisa (Krissinel and Henrick, 2007), with images produced using the resulting PDB file generated by Pisa. Further dimer interfaces with a high Complex Significance Score were identified and analysed.

### 4.10 Modelling

Models of the WT and variant structures of domains Fn3-11, Fn3-20, Fn3-49, Fn3-85 and Fn3-90 were generated using Alphafold2 (Jumper et al., 2021) via the ColabFold: AlphaFold2 w/MMSeqs2 applet with Amber relaxation (Mirdita et al., 2022), with their clashscores calculated using SWISS-MODEL (Waterhouse et al., 2018).

### 4.11 Bioinformatic missense variant analysis

For each missense variant, mCSM and Polyphen-2 values were retrieved from TITINdb (Laddach et al., 2017). SIFT scores were retrieved from the online server (https://sift.bii.a-star.edu.sg) following input of the domain the missense variant resides in. CADD scores were retrieved using the SNV lookup table on the online server (https://cadd.bihealth.org). Gibbs free energy change upon mutation was calculated using the Stability command in FoldX 5 after energy minimization of the models using the RepairPDB command. For WT domains, the crystal structures were used as the input models. For Fn3-3 and Fn3-56, the variant crystal structures were used and for all other variant domains the mutation was introduced into the WT crystal structure in PyMol, with the rotamer that produced the least clashes selected.

### 4.12 Molecular graphics

All images of protein structures were generated using PyMol Molecular Graphics System, version 2.3.0 (Schrodinger, LLC).

## Funding

MR, RN and AA were supported by the 10.13039/501100000274British Heart Foundation grant RG/15/8/31480. MP was supported by the 10.13039/501100000274British Heart Foundation grant PG/15/22/31360. LRL was supported by an UKRI MRC CARP award MR/T005181/1. MG holds the BHF Chair of Molecular Cardiology.

## PDB Deposition

Crystal structures and accompanying data have been deposited to the Protein Data Bank with accession codes 4o00 (Fn3-3), 8OIY (Fn3-3 Arg15908His), 8OS3 (Fn3-11), 8OMW (Fn3-20), 8OSD (Fn3-49), 8OQ9 (Fn3-56), 8ORL (Fn3-56 Ser22326Gly), 8OTS (Fn3-85) and 8OTY (Fn3-90).

## CRediT authorship contribution statement

**Martin Rees:** Investigation, Writing – original draft. **Roksana Nikoopour:** Investigation, Writing – review & editing. **Alexander Alexandrovich:** Investigation, Resources. **Mark Pfuhl:** Investigation, Writing – review & editing. **Luis R. Lopes:** Resources. **Mohammed M. Akhtar:** Resources. **Petros Syrris:** Resources. **Perry Elliott:** Resources. **Gerry Carr-White:** Resources. **Mathias Gautel:** Conceptualization, Supervision, Funding acquisition, Writing – review & editing.

## Declaration of Competing Interest

The authors declare that they have no known competing financial interests or personal relationships that could have appeared to influence the work reported in this paper.

## Data Availability

Data will be made available on request.
