# Supplementary material for: Structure determination and analysis of titin A-band fibronectin type III domains provides insights for disease-linked variants and protein oligomerisation
Source: J Struct Biol. 2023 Sep;215(3):108009. doi: 10.1016/j.jsb.2023.108009 (PMC10862085; doi:10.1016/j.jsb.2023.108009)
Supplement: Supplementary data 1 [file mmc1.pdf]

Supplementary Figures and Tables for Rees *et al* 2023

```
Fn3-127   ---GSPNSPEGPLEYDDIQVRSVRVSWRPPADDGGADILGYILERR-----EVP-K--AAWYTID----SRVRG-T----SLVVK-GLKEN-VEYHFRVSAENQFG-ISKPLKSEEPVTPKTPLN-   102
Fn3-130   ---DKPDKPTGPIVIEALLKNSAVISWKPPADDGGSWITNYVVEKC-----EAKEG--AEWQLVS----SAISV-T----TCRIV-NLTEN-AGYYFRVSAQNTFG-ISDPLEVSSVVIIKSPFE-   103
Fn3-108   ---GPPSKPKGPIRFDEIKADSVILSWDVPEDNGGGEITCYSIEKR-----ETS-Q--TNWKMVC----SSVAR-T----TFKVP-NLVKD-AEYQFRVRAENRYG-VSQPLVSSI-IVAKHQFR-   101
Fn3-34    ---DVPGPPTGPINILDVTPEHMTISWQPPKDDGGSPVINYIVEKQ-----DTR-K--DTWGVVS----SGSSK-T----KLKIP-HLQKG-CEYVFRVRAENKIG-VGPPLDSTP-TVAKHKFS-   101
Fn3-116   ---GRPGPVTGPIEVSSVSAESCVLSWGEPKDGGGTEITNYIVEKR-----ESG-T--TAWQLVN----SSVKR-T----QIKVT-HLTKY-MEYSFRVSSENRFG-VSKPLESAP-IIAEHPFV-   101
Fn3-29    ---DRPGPPVGPVSFDEVTKDYMVISWKPPLDDGGSKITNYIIEKK-----EVG-K--DVWMPVT----SASAK-T----TCKVS-KLLEG-KDYIFRIHAENLYG-ISDPLVSDS-MKAKDRFR-   101
Fn3-24    ---GRPGPPVGPIKFESVSADQMTLSWFPPKDDGGSKITNYVIEKR-----EAN-R--KTWVHVS----SEPKE-C----TYTIP-KLLEG-HEYVFRIMAQNKYG-IGEPLDSEP-ETARNLFS-   101
Fn3-52    ---GKPGIPTGPIKFDEVTAEAMTLKWAPPKDDGGSEITNYILEKR-----DSV-N--NKWVTCA----SAVQK-T----TFRVT-RLHEG-MEYTFRVSAENKYG-VGEGLKSEP-IVARHPFD-   101
Fn3-124   ---DKPGPPTGPIKIDEIDATSITISWEPPELDGGAPLSGYVVEQR-----DAH-R--PGWLPVS----ESVTR-S----TFKFT-RLTEG-NEYVFRVAATNRFG-IGSYLQSEV-IECRSSIR-   101
Fn3-100   ---DRPGPPTGPVVISDITEESVTLKWEPPKYDGGSQVTNYILLKR-----ETS-T--AVWTEVS----ATVAR-T----MMKVM-KLTTG-EEYQFRIKAENRFG-ISDHIDSAC-VTVKLPYT-   101
Fn3-60    ---DIPGPPTGPIKFDEVSSDFVTFSWDPPENDGGVPISNYVVEMR-----QTD-S--TTWVELA----TTVIR-T----TYKAT-RLTTG-LEYQFRVKAQNRYG-VGPGITSAC-IVANYPFK-   101
Fn3-92    ---DRPGPP-GPIRIDEVSCDSITISWNPPEYDGGCQISNYIVEKK-----ETT-S--TTWHIVS----QAVAR-T----SIKIV-RLTTG-SEYQFRVCAENRYG-KSSYSESSA-VVAEYPFS-   100
Fn3-68    ---DKPGPPTGPVKMDEVTADSITLSWGPPKYDGGSSINNYIVEKR-----DTS-T--TTWQIVS----ATVAR-T----TIKAC-RLKTG-CEYQFRIAAENRYG-KSTYLNSEP-TVAQYPFK-   101
Fn3-76    ---DKPDPPKGPVKFDDVSAESITLSWNPPLYTGGCQITNYIVQKR-----DTT-T--TVWDVVS----ATVAR-T----TLKVT-KLKTG-TEYQFRIFAENRYG-QSFALESDP-IVAQYPYK-   101
Fn3-84    ---EKPGPPVGPVRFDEVSADFVVISWEPPAYTGGCQISNYIVEKR-----DTT-T--TTWHMVS----ATVAR-T----TIKIT-KLKTG-TEYQFRIFAENRYG-KSAPLDSKA-VIVQYPFK-   101
Fn3-37    ---DRPGPC-QNLKVTNVTKENCTISWENPLDNGGSEITNFIVEYR-----KPN-Q--KGWSIVA----SDVTK-R----LIKAN-LL-AN-NEYYFRVCAENKVG-VGPTIETKTPILAINPID-   100
Fn3-1     ---DVPGPV-RNLEVTETFDGEVSLAWEEPLTDGGSKIIGYVVERR-----DIK-R--KTWVLAT----DRAES-C----EFTVT-GLQKGGVEYLFRVSARNRVG-TGEPVETDNPVEARSKYD-   102
Fn3-32    ---DKPGPP-RDLEVSEIRKDSCYLTWKEPLDDGGSVITNYVVERR-----DVA-S--AQWSPLS----ATSKK-K----SHFAK-HLNEG-NQYLFRVAAENQYG-RGPFVETPKPIKALDPLH-   101
Fn3-12    ---GLPGPC-KDIKASDITKSSCKLTWEPPEFDGGTPILHYVLERR-----EAG-R--RTYIPVM----SGENK-L----SWTVK-DLIPN-GEYFFRVKAVNKVG-GGEYIELKNPVIAQDPK--   100
Fn3-4     ---ARPSAP-KELKFGDITKDSVHLTWEPPDDDGGSPLTGYVVEKR-----EVS-R--KTWTKVM----DFVTD-L----EFTVP-DLVQG-KEYLFKVCARNKCG-PGEPAYVDEPVNMSTPAT-   101
Fn3-114   ---DTPGPP-GPITFKDVTRGSATLMWDAPLLDGGARIHHYVVEKR-----EAS-R--RSWQVIS----EKCTR-Q----IFKVN-DLAEG-VPYYFRVSAVNEYG-VGEPYEMPEPIVATEQPA-   101
Fn3-42    ---DTPGPV-SDLKVSDVTKTSCHVSWAPPENDGGSQVTHYIVEKR-----EAD-R--KTWSTVT----PEVKK-T----SFHVT-NLVPG-NEYYFRVTAVNEYG-PGVPTDVPKPVLASDPLS-   101
Fn3-106   ---DTPAAC-QKLQVKHVSRGTVTLLWDPPLIDGGSPIINYVIEKR-----DAT-K--RTWSVVS----HKCSS-T----SFKLI-DLSEK-TPFFFRVLAENEIG-IGEPCETTEPVKAAEVPA-   101
Fn3-122   ---DTPGPC-PSVKVKEVSRDSVTITWEIPTIDGGAPVNNYIVEKR-----EAA-M--RAFKTVT----TKCSK-T----LYRIS-GLVEG-TMYYFRVLPENIYG-IGEPCETSDAVLVSEVPL-   101
Fn3-98    ---DTPGPP-TNITVQDVTKESAVLSWDVPENDGGAPVKNYHIEKR-----EAS-K--KAWVSVT----NNCNR-L----SYKVT-NLQEG-AIYYFRVSGENEFG-VGIPAETKEGVKITEKPS-   101
Fn3-50    ---DTPGPP-VNVTVKEISKDSAYVTWEPPIIDGGSPIINYVVQKR-----DAE-R--KSWSTVT----TECSK-T----SFRVA-NLEEG-KSYFFRVFAENEYG-IGDPGETRDAVKASQTPG-   101
Fn3-82    ---DTPGPP-QNLAVKEVRKDSAFLVWEPPIIDGGAKVKNYVIDKR-----EST-R--KAYANVS----SKCSK-T----SFKVE-NLTEG-AIYYFRVMAENEFG-VGVPVETVDAVKAAEPPS-   101
Fn3-58    ---DTPGPV-LNLRPTDITKDSVTLHWDLPLIDGGSRITNYIVEKR-----EAT-R--KSYSTAT----TKCHK-C----TYKVT-GLSEG-CEYFFRVMAENEYG-IGEPTETTEPVKASEAPS-   101
Fn3-90    ---DSPSAP-VNLTIREVKKDSVTLSWEPPLIDGGAKITNYIVEKR-----ETT-R--KAYATIT----NNCTK-T----TFRIE-NLQEG-CSYYFRVLASNEYG-IGLPAETTEPVKVSEPPL-   101
Fn3-66    ---DTPGPP-QDLKVKEVTKTSVTLTWDPPLLDGGSKIKNYIVEKR-----EST-R--KAYSTVA----TNCHK-T----SWKVD-QLQEG-CSYYFRVLAENEYG-IGLPAETAESVKASERPL-   101
Fn3-74    ---DTPSPP-VNLKVTEITKDSVSITWEPPLLDGGSKIKNYIVEKR-----EAT-R--KSYAAVV----TNCHK-N----SWKID-QLQEG-CSYYFRVTAENEYG-IGLPAQTADPIKVAEVPQ-   101
Fn3-17    ---DVPGPV-LDLKPVVTNRKMCLLNWSDPEDDGGSEITGFIIERK-----DAK-M--HTWRQPI----E-TER-S----KCDIT-GLLEG-QEYKFRVIAKNKFG-CGPPVEIGP-ILAVDPLG-   99
Fn3-19    ---DRPSPP-RNLAVTDIKAESCYLTWDAPLDNGGSEITHYVIDKR-----DAS-RKKAEWEEVT----NTAVE-K----RYGIW-KLIPN-GQYEFRVRAVNKYG-ISDECKSDK-VVIQDPYR-   102
Fn3-27    ---DVPGPVGTPFLAHNLTNESCKLTWFSPEDDGGSPITNYVIEKR-----ESD-R--RAWTPVT----YTVTR-Q----NATVQ-GLIQG-KAYFFRIAAENSIG-MGPFVETSEALVIREPIT-   102
Fn3-14    ---DTPGPP-INFVFEDIRKTSVLCKWEPPLDDGGSEIINYTLEKK-----DKT-KPDSEWIVVT----STLRH-C----KYSVT-KLIEG-KEYLFRVRAENRFG-PGPPCVSKP-LVAKDPFG-   102
Fn3-6     ---DKPGPP-AAFDITDVTNESCLLTWNPPRDDGGSKITNYVVERR-----ATD-S--EVWHKLS----STVKD-T----NFKAT-KLIPN-KEYIFRVAAENMYG-VGEPVQASP-ITAKYQFD-   100
Fn3-44    ---DAPGPPQPPFDISDIDADACSLSWHIPLEDGGSNITNYIVEKC-----DVS-R--GDWVTAL----ASVTK-T----SCRVG-KLIPG-QEYIFRVRAENRFG-ISEPLTSPK-MVAQFPFG-   101
Fn3-39    ---DKPGPV-RNLKIVDVSSDRCTVCWDPPEDDGGCEIQNYILEKC-----ETK-R--MVWSTYS----ATVLT-P----GTTVT-RLIEG-NEYIFRVRAENKIG-TGPPTESKP-VIAKTKYD-   100
Fn3-47    ---DKPGPP-ASVKINKMYSDRAMLSWEPPLEDGGSEITNYIVDKR-----ETS-R--PNWAQVS----ATVPI-T----SCSVE-KLIEG-HEYQFRICAENKYG-VGDPVFTEP-AIAKNPYD-   100
Fn3-111   ---DTPGKVVGPIRFTNITGEKMTLWWDAPLNDGCAPITHYIIEKR-----ETS-R--LAWALIE----DKCEA-Q----SYTAI-KLING-NEYQFRVSAVNKFG-VGRPLDSDP-VVAQIQYT-   101
Fn3-119   ---DSPGPC-GKLTVSRVTQEKCTLAWSLPQEDGGAEITHYIVERR-----ETS-R--LNWVIVE----GECPT-L----SYVVT-RLIKN-NEYIFRVRAVNKYG-PGVPVESEP-IVARNSFT-   100
Fn3-55    ---DVPGPP-GPVEISNVSAEKATLTWTPPLEDGGSPIKSYILEKR-----ETS-R--LLWTVVS----EDIQS-C----RHVAT-KLIQG-NEYIFRVSAVNHYG-KGEPVQSEP-VKMVDRFG-   100
Fn3-103   ---DKPGPPGGPIEFKTVTAEKITLLWRPPADDGGAKITHYIVEKR-----ETS-R--VVWSMVS----EHLEE-C----IITTT-KIIKG-NEYIFRVRAVNKYG-IGEPLESDS-VVAKNAFV-   101
Fn3-95    ---DKPGPPAGPLEINGLTAEKCSLSWGRPQEDGGADIDYYIVEKR-----ETS-H--LAWTICE----GELQM-T----SCKVT-KLLKG-NEYIFRVTGVNKYG-VGEPLESVA-IKALDPFT-   101
Fn3-87    ----RPGPPEGPLKVTGVTAEKCYLAWNPPLQDGGANISHYIIEKR-----ETS-R--LSWTQVS----TEVQA-L----NYKVT-KLLPG-NEYIFRVMAVNKYG-IGEPLESGP-VTACNPYK-   100
Fn3-63    ---DRPGPPEGPLAVTEVTSEKCVLSWFPPLDDGGAKIDHYIVQKR-----ETS-R--LAWTNVA----SEVQV-T----KLKVT-KLLKG-NEYIFRVMAVNKYG-VGEPLESEP-VLAVNPYG-   101
Fn3-71    ---DRPGPPEGPVVISGVTAEKCTLAWKPPLQDGGSDIINYIVERR-----ETS-R--LVWTVVD----ANVQT-L----SCKVT-KLLEG-NEYTFRIMAVNKYG-VGEPLESEP-VVAKNPFV-   101
Fn3-79    ---DRPGPPEGPVQVTGVTSEKCSLTWSPPLQDGGSDISHYVVEKR-----ETS-R--LAWTVVA----SEVVT-N----SLKVT-KLLEG-NEYVFRIMAVNKYG-VGEPLESAP-VLMKNPFV-   101
Fn3-38    ---DRPGEP-ENLHIADKGKTFVYLKWRRPDYDGGSPNLSYHVERRL--------KGSDDWERVH-K--GSIKE-T----HYMVD-RCVEN-QIYEFRVQTKNEGG-ESDWVKTEEVVVKEDLQK-   102
Fn3-28    ---TVPERP-EDLEVKEVTKNTVTLTWNPPKYDGGSEIINYVLESRL--------IGTEKFHKVT-N--DNLLS-R----KYTVK-GLKEG-DTYEYRVSAVNIVG-QGKPSFCTKPITCKDELA-   102
Fn3-107   ---EVPAPI-RDLSMKDSTKTSVILSWTKPDFDGGSVITEYVVERKG--------KGEQTWSHAG-I----SKT-C----EIEVS-QLKEQ-SVLEFRVFAKNEKG-LSDPVTI-GPITVKELII-   99
Fn3-97    ---FLPSPP-SKPKIVDSGKTTITIAWVKPLFDGGAPITGYTVEYKK--------SDDTDWKTSI-Q--S-LRG-T----EYTIS-GLTTG-AEYVFRVKSVNKVG-ASDPSDSSDPQIAKEREE-   101
Fn3-123   ---EVPLVP-AKLEVVDVTKSTVTLAWEKPLYDGGSRLTGYVLEACK--------AGTERWMKVV-T--LKPTV-L----EHTVT-SLNEG-EQYLFRIRAQNEKG-VSEPRETVTAVTVQDLRV-   102
Fn3-8     ---DPPWPP-GKPTVKDVGKTSVRLNWTKPEHDGGAKIESYVIEMLK--------TGTDEWVRVA-E--G-VPT-T----QHLLP-GLMEG-QEYSFRVRAVNKAG-ESEPSEPSDPVLCREKLY-   101
Fn3-115   ---EQPAPP-RRLDVVDTSKSSAVLAWLKPDHDGGSRITGYLLEMRQ--------KGSDFWVEAG-H----TKQ-L----TFTVE-RLVEK-TEYEFRVKAKNDAG-YSEPREAFSSVIIKEPQI-   100
Fn3-59    ---EAPSPP-DSLNIMDITKSTVSLAWPKPKHDGGSKITGYVIEAQR--------KGSDQWTHIT-T----VKG-L----ECVVR-NLTEG-EEYTFQVMAVNSAG-RSAPRES-RPVIVKEQTM-   99
Fn3-99    ---EKPSPP-EKLGVTSISKDSVSLTWLKPEHDGGSRIVHYVVEALE--------KGQKNWVKCA-V----AKS-T----HHVVS-GLREN-SEYFFRVFAENQAG-LSDPRELLLPVLIKEQLE-   100
Fn3-83    ---EPPSPP-GKVTLTDVSQTSASLMWEKPEHDGGSRVLGYVVEMQP--------KGTEKWSIVA-E----SKV-C----NAVVT-GLSSG-QEYQFRVKAYNEKG-KSDPRVLGVPVIAKDLTI-   100
Fn3-91    ---EPPLPP-GRVTLVDVTRNTATIKWEKPESDGGSKITGYVVEMQT--------KGSEKWSTCT-Q----VKT-L----EATIS-GLTAG-EEYVFRVAAVNEKG-RSDPRQLGVPVIARDIEI-   100
Fn3-67    ---ERPLPP-GKITLMDVTRNSVSLSWEKPEHDGGSRILGYIVEMQT--------KGSDKWATCA-T----VKV-T----EATIT-GLIQG-EEYSFRVSAQNEKG-ISDPRQLSVPVIAKDLVI-   100
Fn3-75    ---EVPQPP-GKITVDDVTRNSVSLSWTKPEHDGGSKIIQYIVEMQA--------KHSEKWSECA-R----VKS-L----QAVIT-NLTQG-EEYLFRVVAVNEKG-RSDPRSLAVPIVAKDLVI-   100
Fn3-16    ---ISPPGP-PIPRVTDTSSTTIELEWEPPAFNGGGEIVGYFVDKQL--------VGTNEWSRC--TEK-MIKV-R----QYTVK-EIREG-ADYKLRVSAVNAAG-EGPPGETQPVTVAEPQE--   101
Fn3-105   ---DPPGPP-AKIRIADSTKSSITLGWSKPVYDGGSAVTGYVVEIRQ--------GEEEEWTTVS-TKG-EVRT-T----EYVVS-NLKPG-VNYYFRVSAVNCAG-QGEPIEMNEPVQAKDILE-   103
Fn3-26    ---IAPPGP-PFPKVTDWTKSSADLEWSPPLKDGGSKVTGYIVEYKE--------EGKEEWEKG--KDK-EVRG-T----KLVVT-GLKEG-AFYKFRVRAVNIAG-IGEPGEVTDVIEMKDRLV-   102
Fn3-43    ---SEPDPP-RKLEVTEMTKNSATLAWLPPLRDGGAKIDGYITSYRE--------EEQPADRWT--EYS-VVKD-L----SLVVT-GLKEG-KKYKFRVAARNAVG-VSLPREAEGVYEAKEQLL-   102
Fn3-113   ---NPPGPP-TVVKVTDTSKTTVSLEWSKPVFDGGMEIIGYIIEMCK--------ADLGDWHKVN-A--EACVK-T----RYTVT-DLQAG-EEYKFRVSAINGAG-KGDSCEVTGTIKAVDRLT-   102
Fn3-31    ---AKPSPP-VNPEAIDTTCNSVDLTWQPPRHDGGSKILGYIVEYQK--------VGDEEWRRAN-HTPESCPE-T----KYKVT-GLRDG-QTYKFRVLAVNAAG-ESDPAHVPEPVLVKDRLE-   104
Fn3-126   ---APPGKP-QNPRVTDTTRTSVSLAWSVPEDEGGSKVTGYLIEMQK--------VDQHEWTKCN-TTPTKI---R----EYTLT-HLPQG-AEYRFRVLACNAGG-PGEPAEVPGTVKVTEMLE-   102
Fn3-18    ---GPPTSP-ERLTYTERTKSTITLDWKEPRSNGGSPIQGYIIEKRR--------HDKPDFERVN-KR--LCPT-T----SFLVE-NLDEH-QMYEFRVKAVNEIG-ESEPSLPLNVVIQDDEVP-   102
Fn3-33    ---HPPGPP-KDLHHVDVDKTEVSLVWNKPDRDGGSPITGYLVEYQE--------EGTQDWIKFK-TVT----N-L----ECVVT-GLQQG-KTYRFRVKAENIVG-LGLPDTTIPIECQEKLV--   99
Fn3-41    ---EPPGPP-TNFRVVDTTKHSITLGWGKPVYDGGAPIIGYVVEMRPKIADA---SPDEGWKRCN-AAA-QLVR-K----EFTVT-SLDEN-QEYEFRVCAQNQVG-IGRPAELKEAIKPKEILE-   108
Fn3-57    ---YPPGPP-SNPHVTDTTKKSASLAWGKPHYDGGLEITGYVVEHQK--------VGDEAWIKDT-TGT-ALRI-T----QFVVP-DLQTK-EKYNFRISAINDAG-VGEPAVIPDVEIVEREMA-   103
Fn3-49    DPQYPPGPP-AFPKVYDTTRSSVSLSWGKPAYDGGSPIIGYLVEVKR--------ADSDNWVRCN-LPQ-NLQK-T----RFEVT-GLMED-TQYQFRVYAVNKIG-YSDPSDVPDKHYPKDILI-   106
Fn3-65    ---FKPGPP-GNPRVLDTSRSSISIAWNKPIYDGGSEITGYMVEIAL--------PEEDEWQIVT-PPA-GLKA-T----SYTIT-GLTEN-QEYKIRIYAMNSEG-LGEPALVPGTPKAEDRML-   103
Fn3-81    ---FKPGPP-TNAHIVDTTKNSITLAWGKPIYDGGSEILGYVVEICK--------ADEEEWQIVT-PQT-GLRV-T----RFEIS-KLTEH-QEYKIRVCALNKVG-LGEATSVPGTVKPEDKLE-   103
Fn3-73    ---YKPGPP-NNPKVIDITRSSVFLSWSKPIYDGGCEIQGYIVEKCD--------VSVGEWTMCT-PPT-GINK-T----NIEVE-KLLEK-HEYNFRICAINKAG-VGEHADVPGPIIVEEKLE-   103
Fn3-89    ---YPPGPP-SNPKVTDTSRSSVSLAWSKPIYDGGAPVKGYVVEVKE--------AAADEWTTCT-PPT-GLQG-K----QFTVT-KLKEN-TEYNFRICAINSEG-VGEPATLPGSVVAQERIE-   103
Fn3-36    ---KPPGPP-INPKLKDKSRETADLVWTKPLSDGGSPILGYVVECQK--------PGTAQWNRIN-KDE-LIRQ-C----AFRVP-GLIEG-NEYRFRIKAANIVG-EGEPRELAESVIAKDILH-   103
Fn3-21    ---FPPGPP-SCPEVKDKTKSSISLGWKPPAKDGGSPIKGYIVEMQE--------EGTTDWKRVN-EPDKLITT-C----ECVVP-NLKEL-RKYRFRVKAVNEAG-ESEPSDTTGEIPATDIQE-   104
Fn3-121   ---YTPGPP-SAPRVVDTTKHSISLAWTKPMYDGGTDIVGYVLEMQE--------KDTDQWYRVH-TNA-TIRN-T----EFTVP-DLKMG-QKYSFRVAAVNVKG-MSEYSESIAEIEPVERIE-   103
Fn3-110   ---SPVDPP-GTPDYIDVTRETITLKWNPPLRDGGSKIVGYSIEKR-----Q----GNERWVRCN-F--TDVSE-C----QYTVT-GLSPG-DRYEFRIIARNAVGTISPPSQSSGIIMTRDENV-   102
Fn3-118   ---NPVDAP-GRPEVTDVTRSTVSLIWSAPAYDGGSKVVGYIIERK-----PVSEVGDGRWLKCN-Y--TIVSD-N----FFTVT-ALSEG-DTYEFRVLAKNAAGVISKGSESTGPVTCRDEYA-   106
Fn3-102   ---DACEPP-RNVRITDISKNSVSLSWQQPAFDGGSKITGYIVERR-----D---LPDGRWTKAS-F--TNVTE-T----QFIIS-GLTQN-SQYEFRVFARNAVGSISNPSEVVGPITCIDSYG-   103
Fn3-54    ---DPIDPP-GKPEVINITRNSVTLIWTEPKYDGGHKLTGYIVEKR-----D---LPSKSWMKAN-H--VNVPE-C----AFTVT-DLVEG-GKYEFRIRAKNTAGAISAPSESTETIICKDEYE-   103
Fn3-94    ---DPCDPP-GQPEVTNITRKSVSLKWSKPHYDGGAKITGYIVERR-----E---LPDGRWLKCN-Y--TNIQE-T----YFEVT-ELTED-QRYEFRVFARNAADSVSEPSESTGPIIVKDDVE-   103
Fn3-62    ---DPIDPP-GKPVPLNITRHTVTLKWAKPEYTGGFKITSYIVEKR-----D---LPNGRWLKAN-F--SNILE-N----EFTVS-GLTED-AAYEFRVIAKNAAGAISPPSEPSDAITCRDDVE-   103
Fn3-78    ---DPCDPP-GTPEPIMVKRNEITLQWTKPVYDGGSMITGYIVEKR-----D---LPDGRWMKAS-F--TNVIE-T----QFTVS-GLTED-QRYEFRVIAKNAAGAISKPSDSTGPITAKDEVE-   103
Fn3-70    ---DPCDPP-GRPEAIIVTRNSVTLQWKKPTYDGGSKITGYIVEKK-----E---LPEGRWMKAS-F--TNIID-T----HFEVT-GLVED-HRYEFRVIARNAAGVFSEPSESTGAITARDEVD-   103
Fn3-86    ---DPCDPP-GRPEAIVITRNNVTLKWKKPAYDGGSKITGYIVEKK-----D---LPDGRWMKAS-F--TNVLE-T----EFTVS-GLVED-QRYEFRVIARNAAGNFSEPSDSSGAITARDEID-   103
Fn3-45    ---GVPSEP-KNARVTKVNKDCIFVAWDRPDSDGGSPIIGYLIERK-----E---RNSLLWVKAN-D--TLVRS-T----EYPCA-GLVEG-LEYSFRIYALNKAG-SSPPSKPTEYVTARMPVD-   102
Fn3-117   ---VPPSAP-TRPEVYHVSANAMSIRWEEPYHDGGSKIIGYWVEKK-----E---RNTILWVKEN-K--VPCLE-C----NYKVT-GLVEG-LEYQFRTYALNAAG-VSKASEASRPIMAQNPVD-   102
Fn3-109   ---RIPGPP-GKPVIYNVTSDGMSLTWDAPVYDGGSEVTGFHVEKK-----E---RNSILWQKVN-T--SPISG-R----EYRAT-GLVEG-LDYQFRVYAENSAG-LSSPSDPSKFTLAVSPVD-   102
Fn3-53    ---DVPDAP-PPPNIVDVRHDSVSLTWTDPKKTGGSPITGYHLEFK-----E---RNSLLWKRAN-K--TPIRM-R----DFKVT-GLTEG-LEYEFRVMAINLAG-VGKPSLPSEPVVALDPID-   102
Fn3-101   ---TTPGPP-STPWVTNVTRESITVGWHEPVSNGGSAVVGYHLEMK-----D---RNSILWQKAN-K--LVIRT-T----HFKVT-TISAG-LIYEFRVYAENAAG-VGKPSHPSEPVLAIDACE-   102
Fn3-61    ---KVPGPP-GTPQVTAVTKDSMTISWHEPLSDGGSPILGYHVERK-----E---RNGILWQTVS-K--ALVPG-N----IFKSS-GLTDG-IAYEFRVIAENMAG-KSKPSKPSEPMLALDPID-   102
Fn3-93    ---SPPGPP-GTPKVVHATKSTMLVTWQVPVNDGGSRVIGYHLEYK-----E---RSSILWSKAN-K--ILIAD-T----QMKVS-GLDEG-LMYEYRVYAENIAG-IGKCSKSCEPVPARD----   99
Fn3-69    ---KVPGPP-GTPVVTLSSRDSMEVQWNEPISDGGSRVIGYHLERK-----E---RNSILWVKLN-K--TPIPQ-T----KFKTT-GLEEG-VEYEFRVSAENIVG-IGKPSKVSECYVARDPCD-   102
Fn3-85    ---KEPGPP-GTPFVTSISKDQMLVQWHEPVNDGGTKIIGYHLEQK-----E---KNSILWVKLN-K--TPIQD-T----KFKTT-GLDEG-LEYEFKVSAENIVG-IGKPSKVSECFVARDPCD-   102
Fn3-77    ---KEPGPP-GTPFATAISKDSMVIQWHEPVNNGGSPVIGYHLERK-----E---RNSILWTKVN-K--TIIHD-T----QFKAQ-NLEEG-IEYEFRVYAENIVG-VGKASKNSECYVARDPCD-   102
Fn3-46    ---MPVDPP-GKPEVIDVTKSTVSLIWARPKHDGGSKIIGYFVEAC-----K---LPGDKWVRCN-TAPHQIPQ-E----EYTAT-GLEEK-AQYQFRAIARTAVN-ISPPSEPSDPVTILAENV-   104
Fn3-128   --NPPEPPS-NPPEVLDVTKSSVSLSWSRPKDDGGSRVTGYYIERK-----E---TSTDKWVRHN-K--TQITT-T----MYTVT-GLVPD-AEYQFRIIAQNDVG-LSETSPASEPVVCKDPFD-   103
Fn3-22    ---DVPGPP-KDLKVSDITRGSCRLSWKMPDDDGGDRIKGYVIEKR-----T---IDGKAWTKVN-P--DCGST------TFVVP-DLLSE-QQYFFRVRAENRFG-IGPPVETIQRTTARDPIY-   101
Fn3-20    ---RLPGPP-GKPKVLARTKGSMLVSWTPPLDNGGSPITGYWLEKR-----E---EGSPYWSRVS-R--APITKVGLKGVEFNVP-RLLEG-VKYQFRAMAINAAG-IGPPSEPSDPEVAGDPIF-   107
Fn3-25    ---SVPGAP-DKPTVSSVTRNSMTVNWEEPEYDGGSPVTGYWLEMK-----D---TTSKRWKRVN-R--DPIKAMT-LGVSYKVT-GLIEG-SDYQFRVYAINAAG-VGPASLPSDPATARDPIA-   106
Fn3-40    ---DKPGRP-DPPEVTKVSKEEMTVVWNPPEYDGGKSITGYFLEKK-----E---KHSTRWVPVN-K--SAIPE-R----RMKVQ-NLLPD-HEYQFRVKAENEIG-IGEPSLPSRPVVAKDPIE-   102
Fn3-15    ---GPPDAP-DKPIVEDVTSNSMLVKWNEPK-DNGSPILGYWLEKR-----E---VNSTHWSRVN-K--SLLNA-L----KANVD-GLLEG-LTYVFRVCAENAAG-PGKFSPPSDPKTAHDPIS-   101
Fn3-30    ---RVPDAP-DQPIVTEVTKDSALVTWNKPH-DGGKPITNYILEKR-----E---TMSKRWARVT-K--DPIHPYT----KFRVP-DLLEG-CQYEFRVSAENEIG-IGDPSPPSKPVFAKDPIA-   102
Fn3-2     ---DVPGPP-LNVTITDVNRFGVSLTWEPPEYDGGAEITNYVIELR-----D---KTSIRWDTAM-T--VRAED-L----SATVT-DVVEG-QEYSFRVRAQNRIG-VGKPSAATPFVKVADPIE-   102
Fn3-10    ---TTPGPP-YALAVVDVTKRHVDLKWEPPKNDGGRPIQRYVIEKK-----E---RLGTRWVKAG-K--TAGPD-C----NFRVT-DVIEG-TEVQFQVRAENEAG-VGHPSEPTEILSIEDPTS-   102
Fn3-51    ---QTPGPV-VDLKVRSVSKSSCSIGWKKPHSDGGSRIIGYVVDFL---------TEENKWQRVM------KSLS-L----QYSAK-DLTEG-KEYTFRVSAENENG-EGTPSEITVV--ARDDVV-   97
Fn3-5     ---TVPDPP-ENVKWRDRTANSIFLTWDPPKNDGGSRIKGYIVERC-----P---RGSDKWVACG-E--PV-AE-T----KMEVT-GLEEG-KWYAYRVKALNRQG-ASKPSRPTEEIQAVDTQE-   101
Fn3-3     ---ERPSPP-VNLTSSDQTQSSVQLKWEPPLKDGGSPILGYIIERC-----E---EGKDNWIRCN-M--KLVPE-L----TYKVT-GLEKG-NKYLYRVSAENKAG-VSDPSEILGPLTADDAFVE   103
Fn3-13    ---QPPDPP-VDVEVHNPTAEAMTITWKPPLYDGGSKIMGYIIEKI-----A---KGEERWKRCN-E--HLVPI-L----TYTAK-GLEEG-KEYQFRVRAENAAG-ISEPSRATPPTKAVDPID-   102
Fn3-11    ---SPPSPP-LDLHVTDAGRKHIAIAWKPPEKNGGSPIIGYHVEMC-----P---VGTEKWMRVN-S--RPIKD-L----KFKVEEGVVPD-KEYVLRVRAVNAIG-VSEPSEISENVVAKDPDCK   104
Fn3-132   ---DVPDPP-RGVKVSDVSRDSVNLTWTEPASDGGSKITNYIVEKC-----A---TTAERWLRVG------QARE-T----RYTVI-NLFGK-TSYQFRVIAENKFG-LSKPSEPSEPTITKEDKT-   100
Fn3-23    ---YPPDPP-IKLKIGLITKNTVHLSWKPPKNDGGSPVTHYIVECLAWDPTG---TKKEAWRQCN-K--RDVEE-L----QFTVE-DLVEG-GEYEFRVKAVNAAG-VSKPSATVGPVTVKDQTC-   107
Fn3-131   ---EKPGAP-GKPTITAVTKDSCVVAWKPPASDGGAKIRNYYLEKR-----E---KKQNKWISVT-T--EEIRE-T----VFSVK-NLIEG-LEYEFRVKCENLGG-ESEWSEISEPITPKSDVP-   102
Fn3-129   ---DKPSQP-GELEILSISKDSVTLQWEKPECDGGKEILGYWVEYR-----Q---SGDSAWKKSN-K--ERIKD-K----QFTIG-GLLEA-TEYEFRVFAENETG-LSRPRRTAMSIKTKLTSG-   102
Fn3-9     ---YPPSPP-RWLEVINITKNTADLKWTVPEKDGGSPITNYIVEKR-----D---VRRKGWQTVD----TTVKD-T----KCTVT-PLTEG-SLYVFRVAAENAIG-QSDYTEIEDSVLAKDTFT-   101
Fn3-120   ----IPSPP-GIPEEVGTGKEHIIIQWTKPESDGGNEISNYLVDKR-----E---KKSLRWTRVNKD--YVVYD-T----RLKVT-SLMEG-CDYQFRVTAVNAAG-NSEPSEASNFISCREPSY-   102
Fn3-48    ---DPPGRC-DPPVISNITKDHMTVSWKPPADDGGSPITGYLLEKR-----E---TQAVNWTKVN-R--KPIIE-R----TLKAT-GLQEG-TEYEFRVTAINKAG-PGKPSDASKAAYARDPQY-   102
Fn3-7     ---DPPGPP-TRLEPSDITKDAVTLTWCEPDDDGGSPITGYWVERL-----D---PDTDKWVRCN-K--MPVKD-T----TYRVK-GLTNK-KKYRFRVLAENLAG-PGKPSKSTEPILIKDPID-   102
Fn3-35    -KFSPPSPP-GKPVVTDITENAATVSWTLPKSDGGSPITGYYMERR-----E---VTG-KWVRVN-K--TPIAD-L----KFRVT-GLYEG-NTYEFRVFAENLAG-LSKPSPSSDPIKACRPIK-   103
Fn3-125   ---RIPGPP-ETLQIFDVSRDGMTLTWYPPEDDGGSQVTGYIVERK-----E---VRADRWVRVN-K--VPVTM-T----RYRST-GLTEG-LEYEHRVTAINARG-SGKPSRPSKPIVAMDPIA-   102
Fn3-112   ---TVPDAP-GIPEPSNITGNSITLTWARPESDGGSEIQQYILERR-----E---KKSTRWVKVISK--RPISE-T----RFKVT-GLTEG-NEYEFHVMAENAAG-VGPASGISRLIKCREPVN-   103
Fn3-104   ---VTPGPP-GIPEVTKITKNSMTVVWSRPIADGGSDISGYFLEKR-----D---KKSLGWFKVL-K--ETIRD-T----RQKVT-GLTEN-SDYQYRVCAVNAAG-QGPFSEPSEFYKAADPID-   102
Fn3-56    ---GPPGPP-EKPEVSNVTKNTATVSWKRPVDDGGSEITGYHVERR-----E---KKSLRWVRAI-K--TPVSD-L----RCKVT-GLQEG-STYEFRVSAENRAG-IGPPSEASDSVLMKDAAY-   102
Fn3-96    ---TVPSPP-TSLEITSVTKESMTLCWSRPESDGGSEISGYIIERR-----E---KNSLRWVRVN-K--KPVYD-L----RVKST-GLREG-CEYEYRVYAENAAG-LSLPSETSPLIRAEDPVF-   102
Fn3-88    ---KPPGPP-STPEVSAITKDSMVVTWARPVDDGGTEIEGYILEKR-----D---KEGVRWTKCN-K--KTLTD-L----RLRVT-GLTEG-HSYEFRVAAENAAG-VGEPSEPSVFYRACDALY-   102
Fn3-72    ---VVPDAP-KAPEVTTVTKDSMIVVWERPASDGGSEILGYVLEKR-----D---KEGIRWTRCH-K--RLIGE-L----RLRVT-GLIEN-HDYEFRVSAENAAG-LSEPSPPSAYQKACDPIY-   102
Fn3-64    ---GPPDPP-KNPEVTTITKDSMVVCWGHPDSDGGSEIINYIVERR-----D---KAGQRWIKCN-K--KTLTD-L----RYKVS-GLTEG-HEYEFRIMAENAAG-ISAPSPTSPFYKACDTVF-   102
Fn3-80    ---VLPGPP-KSLEVTNIAKDSMTVCWNRPDSDGGSEIIGYIVEKR-----D---RSGIRWIKCN-K--RRITD-L----RLRVT-GLTED-HEYEFRVSAENAAG-VGEPSPATVYYKACDPVF-   102
                                   * *  .   :                                                        :   . . .
```

Supplementary Figure 1 (previous page). Clustal Omega alignment of all 132 titin Fn3 domains. 100% conserved, highly conserved and conserved residues indicated by asterisk (*), colon (:) and period (.), respectively. Residues are coloured accordingly: positively charged, magenta; negatively charged, blue; small and/or hydrophobic, red; polar and glycine, green.

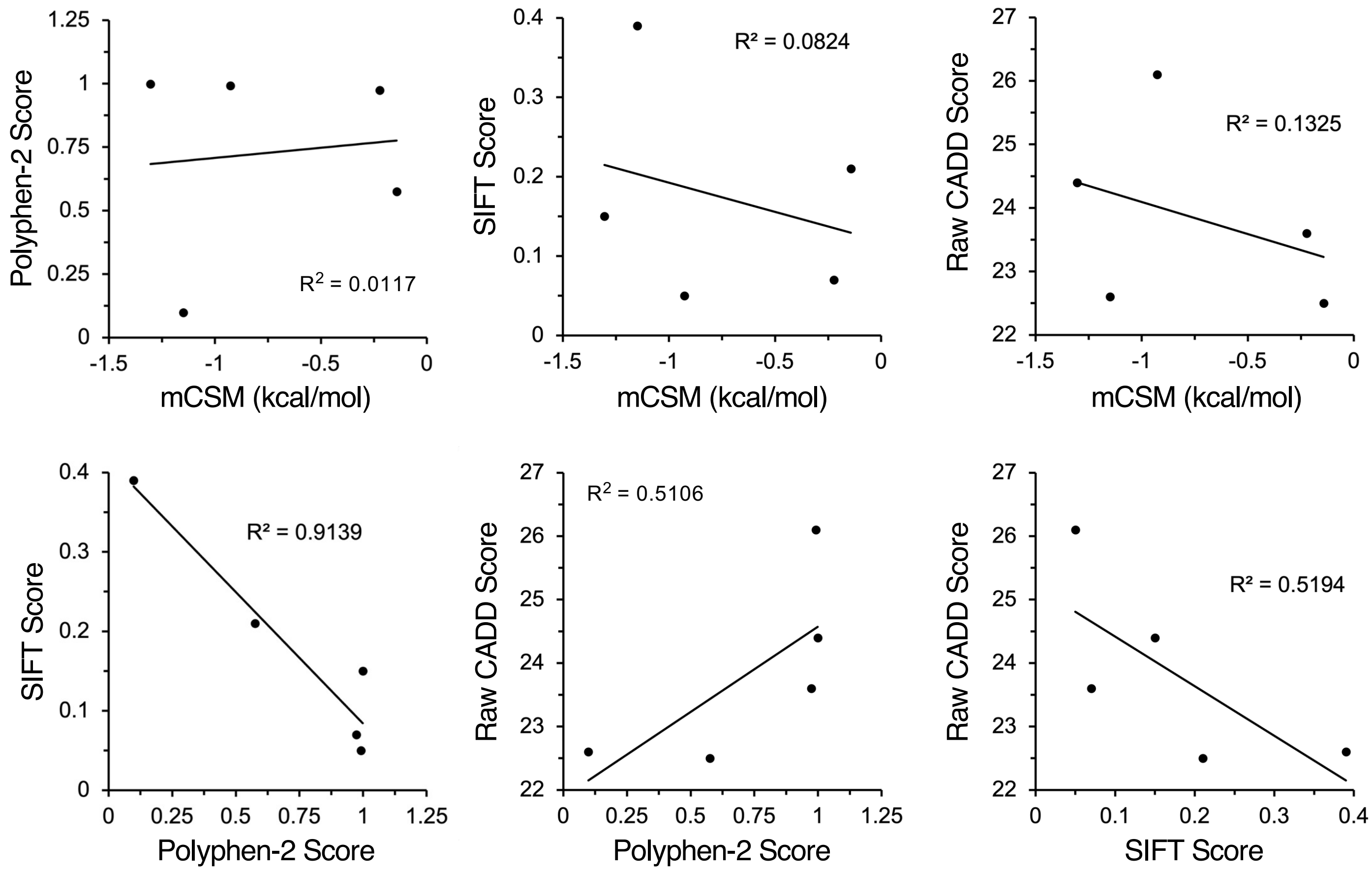

Supplementary Figure 2. Correlations of bioinformatic predictions for the deleterious or destabilising effects of the missense variants in patients with hypertrophic cardiomyopathy analysed in this study. The R^2 goodness-of-fit of the linear trendlines is shown for each plot.

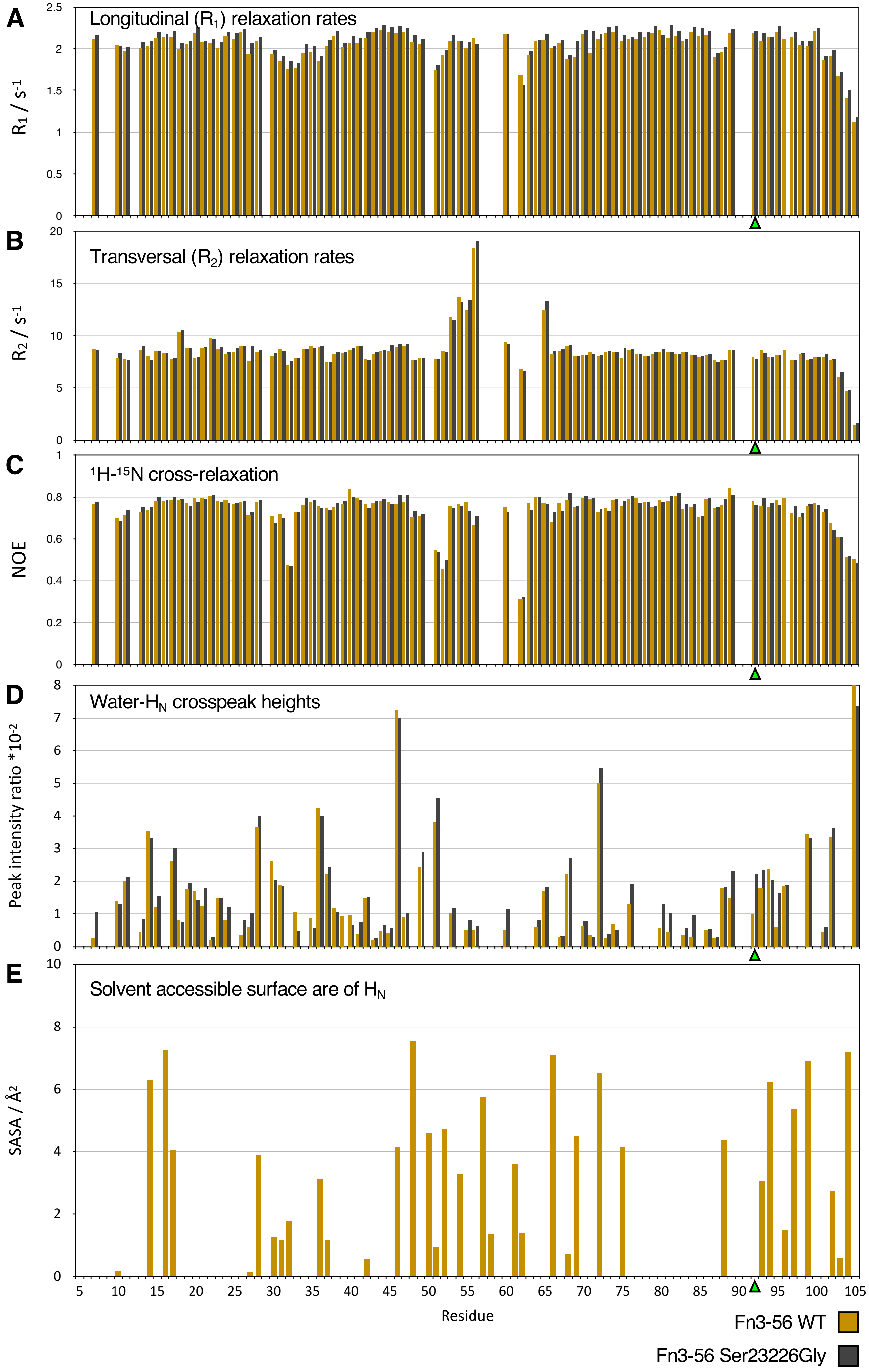
A
Longitudinal (R1) relaxation rates
R1 / s-1
B
Transversal (R2) relaxation rates
R2 / s-1
C
1H-15N cross-relaxation
NOE
D
Water-HN crosspeak heights
Peak intensity ratio *10-2
E
Solvent accessible surface are of HN
SASA / Å2
Residue
Fn3-56 WT
Fn3-56 Ser23226Gly

Supplementary Figure 3 (previous page). Solution dynamics of Fn3-56 WT and Ser23226Gly. A-C: ^{15}N relaxation data. Longitudinal (R_1) **(A)** and transversal (R_2) **(B)** relaxation rates and ^{1}H-^{15}N cross-relaxation **(C)** plotted as function of residue number. **D:** Normalised water-H_N crosspeak heights. High values indicate stronger exchange. **E:** Solvent accessible surface area for all available backbone amide protons in the crystal structure of Fn3-56 WT. Data for WT and Ser23226Gly are shown in mustard and dark grey, respectively. X-axis values are residue numbers in Fn3-56 PDB files. Ser23226Gly corresponds to residue 92 and is indicated on each plot with a green triangle.

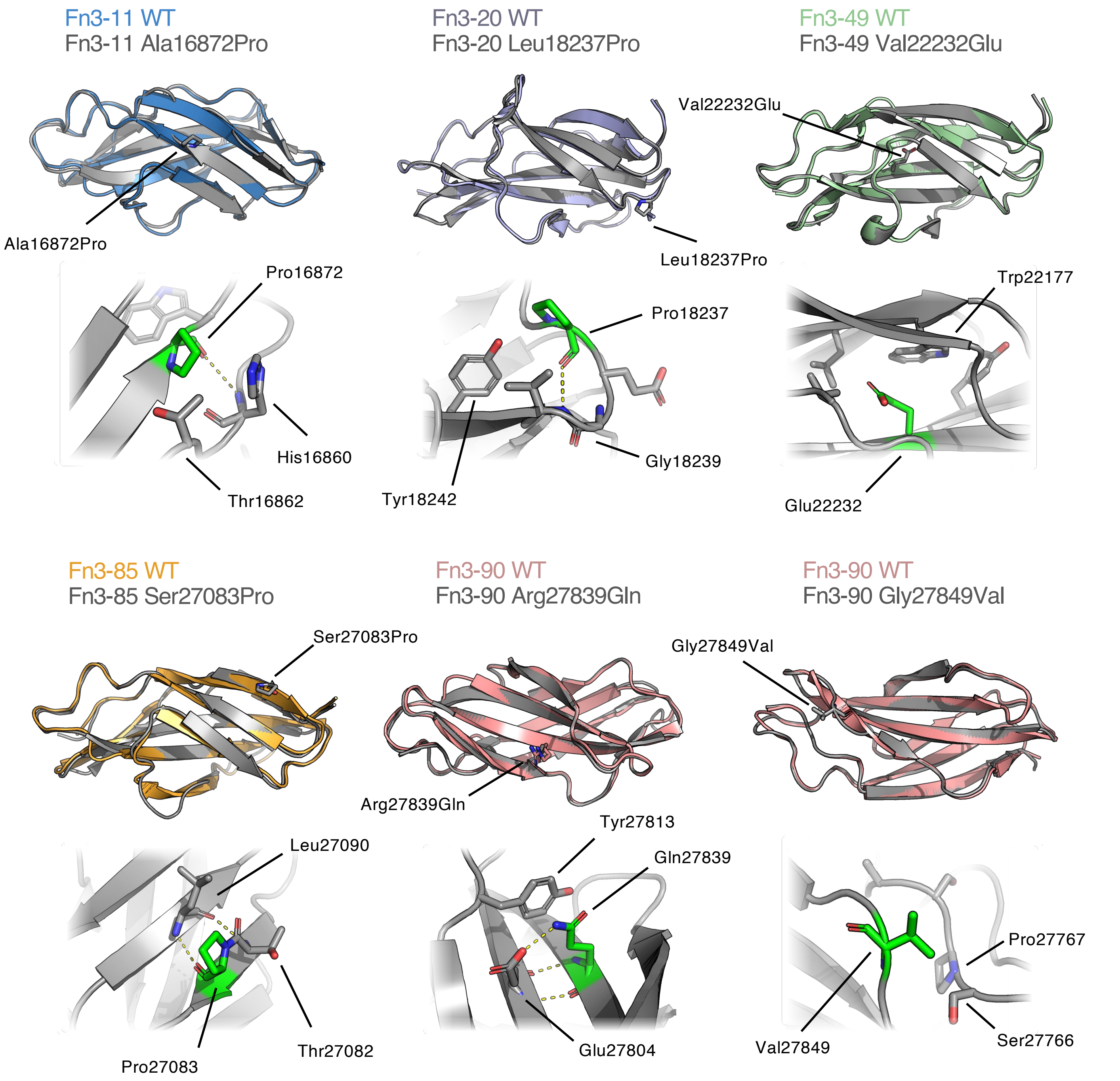

Supplementary Figure 4. Modelling of titin Fn3 domains with variants linked to congenital myopathy and observed in patients with hypertrophic cardiomyopathy. Crystal structures of WT domains (various colours) were aligned with Alphafold2-predicted models of their variant domains (grey), with their N- and C-termini on the left and right, respectively, and the WT and mutated residue shown in stick representation (above). The molecular context of the mutated residue, in green, is shown below in stick representation along side other residues of interest. Hydrogen bonds are shown as dashed yellow lines.

Supplementary Tables

Domain	Start	End
Fn3-3	15858	15960
Fn3-11	16851	16954
Fn3-20	18164	18271
Fn3-49	22152	22257
Fn3-56	23138	23239
Fn3-85	27071	27172
Fn3-90	27765	27865

Supplementary table 1. Domain phasing of constructs used in this work. Residues numbered according to the inferred complete (IC) transcript NM_001267550.

Domain:	Fn3-3 WT	Fn3-3 R15908H	Fn3-11 WT	Fn3-20 WT	Fn3-49 WT
Protein concentration (mg/ml):	10	10	45	42	42
Storage buffer					
Buffer:	10mM Tris-HCl pH 7.5	10mM Tris-HCl pH 7.5	30mM HEPES pH 7.5	10mM Tris-HCl pH 7.5	30mM HEPES pH 7.5
Salt:	50mM NaCl	50mM NaCl	150mM NaCl	50mM NaCl	150mM NaCl
Additives:	0.5mM DTT	0.5mM DTT	1mM DTT	0.5mM DTT	1mM DTT
Crystallisation buffer					
Buffer:	0.1M SPG pH 4.0	0.1M sodium acetate pH 4.5	0.1M PCB pH 9.0	0.1M TBG pH 9.0	N/A
Salt:	N/A	0.2M Lithium sulphate	N/A	N/A	0.1M Potassium thiocyanate
Precipitant:	31% w/v PEG 1500	50% v/v PEG 400	25% w/v PEG 1500	25% w/v PEG 1500	30% w/v PEG MME2000

Domain:	Fn3-56 WT	Fn3-56 S23226G	Fn3-85 WT	Fn3-90 WT
Protein concentration (mg/ml):	11	11	24	15
Storage buffer				
Buffer:	10mM Tris-HCl pH 7.5	10mM Tris-HCl pH 7.5	30mM HEPES pH 7.5	30mM HEPES pH 7.5
Salt:	50mM NaCl	50mM NaCl	150mM NaCl	150mM NaCl
Additives:	0.5mM DTT	0.5mM DTT	1mM DTT	1mM DTT
Crystallisation buffer				
Buffer:	0.1M MES	0.1M MES	0.1M Bis-Tris propane pH 6.5	0.1M Sodium acetate pH 5.0
Salt:	10mM ZnCl2	10mM ZnCl2	0.2M sodium iodide	0.2M CaCl
Precipitant:	20% (w/v) PEG6000	20% (w/v) PEG6000	20% w/v PEG3350	20% w/v PEG6000

Supplementary table 2. Crystallisation conditions for crystal structures determined in this work. SPG is succinic acid, sodium dihydrogenate phosphate and glycine at a 2:7:7 molar ratio. PCB is sodium propionate, sodium cacodylate and BIS-TRIS propane at a 2:1:2 molar ratio. TBG is sodium tartrate dihydrate, BIS-TRIS and glycylglycine at a 3:2:2 molar ratio.

Domain:	Fn3-3 WT	Fn3-3 R15908H	Fn3-11 WT	Fn3-20 WT	Fn3-49 WT
Beamline:	Oxford Xcalbur	Diamond I04	Diamond I02	Diamond I02	Diamond I02
Transmission (%):	100	47	50	20	50
Rotation per frame (º):	0.5	1	0.1	0.15	0.1
No. frames:	204	180	1800	1200	1800
Time per frame (s):	100	0.5	0.04	0.04	0.04

Domain:	Fn3-56 WT	Fn3-56 S23226G	Fn3-85 WT	Fn3-90 WT
Beamline:	Diamond I04	Diamond I02	Diamond I04	Diamond I02
Transmission (%):	50	20	50	60
Rotation per frame (º):	0.1	0.1	0.1	0.1
No. frames:	610	1800	1800	1800
Time per frame (s):	0.05	0.04	0.05	0.1

Supplementary table 3. Data collection information for crystal structures determined in this work.

Variant (Domain):		Arg15908His (Fn3-3)	Ala16872Pro (Fn3-11)	Ser23226Gly (Fn3-56)	Ser27083Pro (Fn3-85)	Arg27839Gln (Fn3-90)
mCSM	kcal/mol:	-1.304	-0.223	-0.927	-0.143	-1.149
	class:	Destabilizing	Destabilizing	Destabilizing	Destabilizing	Destabilizing
Polyphen-2	score:	0.999	0.974	0.992	0.575	0.098
	class:	Probably damaging	Probably damaging	Probably damaging	Possibly damaging	Benign
SIFT	score:	0.15	0.07	0.05	0.21	0.39
	interpretation:	non-deleterious	non-deleterious	deleterious	non-deleterious	non-deleterious
CADD	score:	24.4	23.6	26.1	22.5	22.6
	interpretation:	deleterious	deleterious	deleterious	deleterious	deleterious
ClinVar	rs number:	rs72677237	N/A	rs72646885	rs186273940	rs376820301
	interpretation:	Benign/Likely benin	N/A	Benign/Likely benin	Conflicting (Uncertain/Likely benign)	Conflicting (Benign/Likely benign)

Supplementary table 4. Bioinformatic predictors and ClinVar analysis of effect of missense variants from HCM cohort in this study. For Polyphen-2, scores above 0.908 are classified as "probably damaging", above 0.446 as "possibly damaging" and less than 0.446 as "benign". For SIFT, scores above 0.05 are classified as "non-deleterious" and below as "deleterious". For CADD, scores above 15 are classified as "deleterious".

		RMSD (Å)		Clashscore (model)		Free energy change upon mutation
		WT (crystal structure) vs. WT (model)	WT (crystal structure) vs. Variant (model)			
Domain	Variant			WT	Variant	ΔΔG (kcal/mol)
Fn3-3	Arg15908His	-	-	-	-	1.70
Fn3-11	Ala16872Pro	0.53	0.51	0	1.23	3.04
Fn3-20	Leu18237Pro	0.58	0.67	1.23	1.23	2.09
Fn3-49	Val22232Glu	0.46	0.41	0.63	0.63	4.62
Fn3-56	Ser23226Gly	-	-	-	-	-0.31
Fn3-85	Ser27083Pro	0.78	0.72	1.25	1.87	3.95
Fn3-90	Arg27839Gln	0.56	0.57	0	1.27	-0.12
Fn3-90	Gly27849Val	0.56	0.61	0	1.88	14.40

Supplementary table 5. Bioinformatic predictions of variant effect on domain structure and stability. Structural alignment of WT crystal structures and models used to assess the accuracy of model prediction. Clashscore defined as (number of atoms overlapping by over 0.4A / total number of atoms) x 1000. ΔΔG calculated using FoldX. A positive value for free energy change upon mutation from WT inducates the mutation is predicted to be destabilising; a negative value indicates the mutation is predicted to be stabilising.
